# Sex-specific and age-related progression of auditory neurophysiological deficits in the *Cln3* mouse model of Batten disease

**DOI:** 10.1186/s11689-025-09652-2

**Published:** 2025-11-06

**Authors:** Yanya Ding, Jingyu Feng, Viollandi Prifti, Grace A. Rico, Alexander G. Solorzano, Hayley E. Chang, Edward G. Freedman, John J. Foxe, Kuan Hong Wang

**Affiliations:** Department of Neuroscience, University of Rochester Medical Center, Rochester, NY 14642 USA

**Keywords:** EEG, Auditory evoked potential (AEP), Duration mismatch negativity (MMN), Auditory brainstem response (ABR), Translational biomarker

## Abstract

**Background:**

CLN3 disease, also known as juvenile Batten disease, is a recessively inherited neurodevelopmental disorder caused by mutations in the *CLN3* gene. It represents the most common form of Neuronal Ceroid Lipofuscinoses (NCLs), a group of lysosomal storage disorders that impair brain function. Clinical features include progressive vision loss, language impairment, and cognitive decline. The early onset of visual deficits complicates the neurological assessment of cognitive dysfunction, while the rarity of *CLN3* cases limits the study of sex-specific disease trajectories in humans. Therefore, there is a critical need for objective, translational biomarkers to monitor disease progression and support therapeutic development in preclinical animal models.

**Methods:**

Building on our recent studies in individuals with CLN3 disease, we developed a parallel experimental paradigm using high-density electroencephalography (EEG) in *Cln3* knockout (*Cln3-/-)* mice to longitudinally assess auditory neurophysiological changes. We applied a duration-based mismatch negativity (MMN) paradigm, similar to that used in our human studies, to evaluate automatic detection of auditory pattern changes in male and female mice between 3 and 9 months of age.

**Results:**

Wild-type (WT) mice of both sexes showed robust and stable duration MMN responses across this age range. In contrast, *Cln3-/-* mice showed marked sex- and age-dependent deficits: female mutants displayed persistent MMN deficits, whereas male mutants exhibited early MMN abnormalities that unexpectedly improved with age. Auditory brainstem responses confirmed intact peripheral hearing in *Cln3-/-* mice, indicating a central origin for the observed abnormalities. Further analyses revealed that MMN impairments were driven by age- and sex-specific alterations in auditory evoked potentials to both standard and deviant stimuli.

**Conclusions:**

These findings demonstrate sex- and age-dependent disruptions in central auditory processing in *Cln3-/-* mice and support auditory duration MMN as a sensitive, translational biomarker of brain dysfunction in CLN3 disease. This approach offers a functional, cross-species measure for tracking disease progression and evaluating therapeutic interventions in Batten disease.

**Supplementary Information:**

The online version contains supplementary material available at 10.1186/s11689-025-09652-2.

## Background

Neuronal ceroid lipofuscinoses (NCLs), or Batten diseases, are a group of recessively inherited neurodevelopmental lysosomal storage disorders (LSDs) that primarily affect children and adolescents [1]. CLN3 disease, also referred to as juvenile Batten disease, results from mutations in the *ceroid lipofuscinosis neuronal 3 (CLN3)* gene [2]. It is characterized by the accumulation of ceroid lipofuscin within lysosomes across various cell types, particularly neurons [3]. Clinical symptoms of CLN3 disease typically emerge around 4–7 years of age, beginning with progressive vision loss, followed by cognitive decline, seizures, motor impairment, and speech and language problems, ultimately leading to premature death, often around the age of 20 [4–6]. Notably, female CLN3 patients tend to experience a later onset of symptoms compared to males but show more rapid disease progression and earlier mortality [7]. The early onset of vision loss, followed by cognitive decline, complicates the use of standard vision-based neurological evaluations and cognitive assessments for disease monitoring. Therefore, there is a critical need for an objective neurophysiological biomarker to track disease progression and evaluate therapeutic outcomes in CLN3 disease.

To develop such a biomarker, previous work in our lab employed electroencephalography (EEG) to measure auditory evoked potentials (AEPs) through the duration mismatch negativity (MMN) paradigm. This approach assesses auditory change detection, a process critical for language comprehension and cognition [8], both of which are impaired in CLN3 patients [9]. Since the peripheral auditory system remains relatively intact in CLN3 patients [10], AEPs — neural responses time-locked to auditory events — provide valuable insights into the integrity of central auditory processing [11, 12]. Auditory duration MMN is elicited by occasionally presenting deviant stimuli with a different duration within a sequence of standard stimuli [13]. Brima and colleagues reported robust auditory duration MMN responses in neurotypical controls, whereas CLN3 patients displayed significantly reduced auditory duration MMN, suggesting impairments in auditory deviant discrimination and auditory sensory memory processes [9]. While the MMN paradigm is a powerful tool for assessing the integrity of sensory processing [14], its application in studying age- and sex-dependent pathophysiological differences in human CLN3 research is constrained by the low prevalence of this genetic disorder [15]. Furthermore, the neurophysiological mechanisms underlying auditory MMN dysfunction in CLN3 disease remain unknown.

To establish a common endophenotype with patients for mechanistic studies, we adopted the auditory duration MMN paradigm in the *Cln3-/-* mouse model. Mice were chosen because their AEPs [11] and MMN responses [16] are characteristically similar to those in humans, while also providing a controlled genetic system. The *Cln3*-/- mouse model used in this study was generated using a knockout strategy that deletes the start codon and the first six exons of the *Cln3* gene, resulting in a complete null allele [17]. This model has been widely used to investigate the consequences of complete CLN3 protein loss in CLN3 disease [18–20]. It recapitulates the primary pathological hallmark of ceroid lipofuscin accumulation and shows evidence of age-dependent neurodegeneration [18]. Ex vivo recordings [21] and in vitro cell culture studies [22] have identified synaptic transmission dysfunctions in the *Cln3-/-* mouse model associated with behavioral deficits. However, existing animal studies lack neurophysiological measures directly applicable to and comparable with human studies, making it difficult to evaluate the clinical relevance of experimental findings in the *Cln3-/-* mouse model.

EEG recordings using the auditory duration MMN paradigm provide a valuable approach for characterizing age and sex-dependent differences in auditory processing in the *Cln3-/-* mouse model. To date, auditory processing deficits in *Cln3* mouse models remain largely uncharacterized, as most prior studies have focused on visual, motor, and cognitive impairments [23–25]. Although human studies have suggested a more severe disease course in female CLN3 patients [26], sex-specific differences of neurophysiological responses in the *Cln3-/-* mouse model have not been thoroughly investigated, largely due to the historical underrepresentation of female subjects in preclinical research [27]. Moreover, existing reports of sex-dependent behavioral and motor deficits across different *Cln3* mouse models have been limited and inconsistent [20, 28, 29]. These gaps underscore the pressing need to apply EEG with the MMN paradigm as a consistent and translatable method for evaluating auditory neurophysiological responses in both sexes of the *Cln3-/-* mouse model.

In this study, surface EEG electrode arrays, comparable to those used in human studies, were implanted on *Cln3-/-* mice and their wild-type (WT) littermates, enabling longitudinal recordings from the same animals 3–9 months of age using the auditory duration MMN paradigm. *Cln3-/-* mice exhibited prominent auditory duration MMN deficits compared to robust responses in WT mice. To ensure that these deficits were not due to peripheral hearing loss, auditory brainstem responses (ABRs), an objective measure of peripheral auditory processing [30, 31], were measured. These assessments confirmed that *Cln3-/-* mice showed no peripheral hearing deficits compared to WT mice at the ages tested for MMN. Furthermore, age- and sex-specific deficits in central AEPs that underlie the MMN deficits in *Cln3-/-* mice were revealed. Together, these findings support the use of auditory duration MMN as a translational neurophysiological biomarker for Batten disease.

## Methods

### Animals

All animal procedures were conducted in accordance with the National Institutes of Health (NIH) Guide for the Care and Use of Laboratory Animals and were approved by the University Committee on Animal Resources (UCAR) at the University of Rochester Medical Center. *Cln3-/-* mice and their age-matched WT littermates of both sexes were obtained from an in-house breeding colony, derived from a line originally sourced from the Jackson Laboratory (B6.129S6-Cln3tm1Nbm/J, JAX:029471, ME). The *Cln3-/-* mouse strain was originally generated on the 129S6 genetic background [17], and subsequently back-crossed into the C57BL/6J background for 10 generations prior to being deposited at the Jackson Laboratory. Mice were genotyped through Transnetyx (Cordova, TN), using a real-time PCR-based assay designed according to the strain-specific genotyping protocol provided by the Jackson Laboratory. All animals had unlimited access to food and water and were housed in a temperature and humidity-controlled environment with a 12–12 h light-dark cycle. No signs of distress were observed in mice during and after EEG or ABR recordings.

### Surgery procedures

Animals were anesthetized with 5% isoflurane in medical oxygen positioned on a stereotactic frame. They were immediately transferred to a heating pad that maintained temperature around 37 °C with 1.5% isoflurane administered through a nose mask at an oxygen flow rate of 2 L/min. Then, Meloxicam (1 mg/kg) was injected subcutaneously (SC), and Pivetal Artificial Tears (Lubricant Ophthalmic Ointment, Pivetal) were applied to the animal’s eyes. Following preparation, a midline incision was made on the mouse’s scalp. To ensure tight contact of the electrode array to the skull, connective tissue was gently scraped away. In addition, 3% hydrogen peroxide (H_2_O_2_) was applied to remove any membrane residue on the mouse skull. The positioning of the skull was verified by leveling bregma and lambda. Holes for anchoring (right occipital), reference (left occipital) and grounding (frontal) electrode contacts were drilled, followed by immediate insertion of screws. A 32-channel electrode array (H32, NeuroNexus Technologies Inc., MI) was placed on the mouse skull with the middle cross sign positioned at the bregma with a drop of sterilized saline for better electrode attachment to the skull. After the electrode array was correctly placed and excess saline outside the electrode contacts was air dried, Metabond (Parkell C&B, Parkell Inc., NY) was applied to cover the electrode array. Dental acrylic cement was also applied to cover the rest of the exposed skull area and secure the connector to the skull. Incised skin was then sutured, and antibiotic ointment was applied to the area of the sutures and dental cement. Animals were carefully monitored at least once a day for seven consecutive days and analgesics were administered orally or subcutaneously, if necessary.

### Acoustic stimulation

Acoustic stimuli were generated using RZ6 Multi-I/O processor with Synapse software (Tucker-Davis Technologies, FL) and presented through a speaker (MF1 Multi-Field Magnetic Speaker, Tucker-Davis Technologies, FL), located 10 cm in front of the animal. Sound pressure level (SPL) was modified using programmable attenuators in the TDT system (Tucker-Davis Technologies, FL). Speaker output was calibrated to 80dB SPL at the position of the ears of the mouse in a soundproof recording chamber. Noise burst stimuli were presented at 80dB SPL in blocks of 1000 trials. 850 trials of standard stimuli (50ms in duration) and 150 trials of deviant stimuli (100ms in duration) were played in a pseudo-random manner, with a constant 400ms interstimulus interval (ISI) in each block.

### EEG data acquisition and analysis

Following surgery recovery, animals were habituated in the soundproof recording chamber without acoustic stimulation for at least 3 days. Electrophysiological recording was not performed during habituation. Animals were head-fixed and placed on a rotating disc with a digital head stage connected. Each habituation session lasted 20 min. Impedance tests were run during habituation sessions to verify the quality of electrode implantation. Animals with electrode impedance smaller than 30 kΩ or greater than 200 kΩ at a test frequency of 1526 Hz were excluded from the study.

For electrophysiological recording, EEG signals were recorded with an RZ10X Expanded Lux-IO Processor (Tucker-Davis Technologies, FL) at a sampling rate of 1000 Hz. Signals were first digitized on the mouse head stage and then filtered during recording by the Synapse software (Tucker-Davis Technologies, FL). Raw data were further processed and analyzed by customized scripts in MATLAB (Mathworks, MA). The applied bandpass filter was 0.1∼100 Hz with the notch filter at 60 Hz.

11 male WT mice, 8 male *Cln3-/-* mice, 6 female WT mice, and 7 female *Cln3-/-* mice were recorded longitudinally from 3 to 9 months of age. Three 5 months old male WT mice and two 5 months old male *Cln3-/-* mice, used for pilot study, were included in addition to longitudinally recorded animals. There were 3 to 5 recording sessions for each animal per age point. Data was first averaged at individual animal level then grouped to compute genotype, age, and sex differences.

Auditory duration MMN was calculated by subtracting AEP to standard stimuli from AEP to deviant stimuli (i.e., MMN = Deviant AEP – Standard AEP). Auditory duration MMN amplitude was quantified within 160-180ms after stimulus onset, the period during which MMN is maximal in all WT mice. Auditory duration MMN differences were also compared between sex- and age-matched WT and *Cln3-/-* mice across all the electrodes and time points, generating topographical maps of these differences (WT MMN – *Cln3-/-* MMN) using 20ms time bins. In addition, similar temporospatial analyses were performed for AEPs to examine whether MMN alterations originate from changes in auditory responses to standard stimuli or deviant stimuli.

### ABR data acquisition and analysis

Mice were anesthetized with ketamine (100 mg/kg) and xylazine (10 mg/kg) by intraperitoneal injection (IP). Body temperature was maintained at 37 °C by a heating pad. Responses were recorded through subcutaneous needle electrodes placed at the vertex (active), ventrolateral to the left ear (reference), and ventrolateral to the right ear (grounding). Acoustic stimuli were generated by the RZ6 Multi-I/O processor with BioSigRZ Software (Tucker-Davis Technologies, FL) and presented through a speaker (MF1 Multi-Field Magnetic Speaker, Tucker-Davis Technologies, FL), positioned 10 cm from the animal’s ear. Speaker output was calibrated by BioSigRZ software (Tucker-Davis Technologies, FL) with a TDT microphone, placed at the same distance as the mouse’s ear. Decreasing sound pressure levels from 90dB SPL to 10dB SPL in steps of 5dB SPL were employed for ABR stimulation. 512 stimuli, presented at the rate of 21 per second, were recorded for each sound level. 18 male WT mice (4 at 3-month-old, 7 at 5-month-old, 4 at 7-month-old, and 3 at 9-month-old), 20 male *Cln3-/-* mice (6 at 3-month-old, 6 at 5-month-old, 4 at 7-month-old, and 4 at 9-month-old), 16 female WT mice (4 at 3-month-old, 4 at 5-month-old, 4 at 7-month-old, and 4 at 9-month-old), and 18 female *Cln3-/-* mice (5 at 3-month-old, 4 at 5-month-old, 5 at 7-month-old, and 4 at 9-month-old) were used for ABR under anesthesia. The ABR procedure lasted 10 min per animal. All animals fully recovered after anesthesia.

ABR data was then processed and analyzed by BioSigRZ Software with a 10ms post-stimulus window. The applied bandpass filter was 300∼30,000 Hz. The notch filter was 60 Hz. The gain was 1 (matched the gain of the preamplifier: Medusa4Z), and the sampling frequency was 12 kHz. Hearing thresholds were determined by visual inspection according to the consensus of four researchers in BioSigRZ Software. Each researcher evaluated raw ABR waveforms individually and determined hearing threshold as the lowest sound level where a waveform could be observed. Researchers were blinded for the age, sex, and genotype of animals they evaluated to avoid bias.

### Statistical analysis

**Fig. 1:**
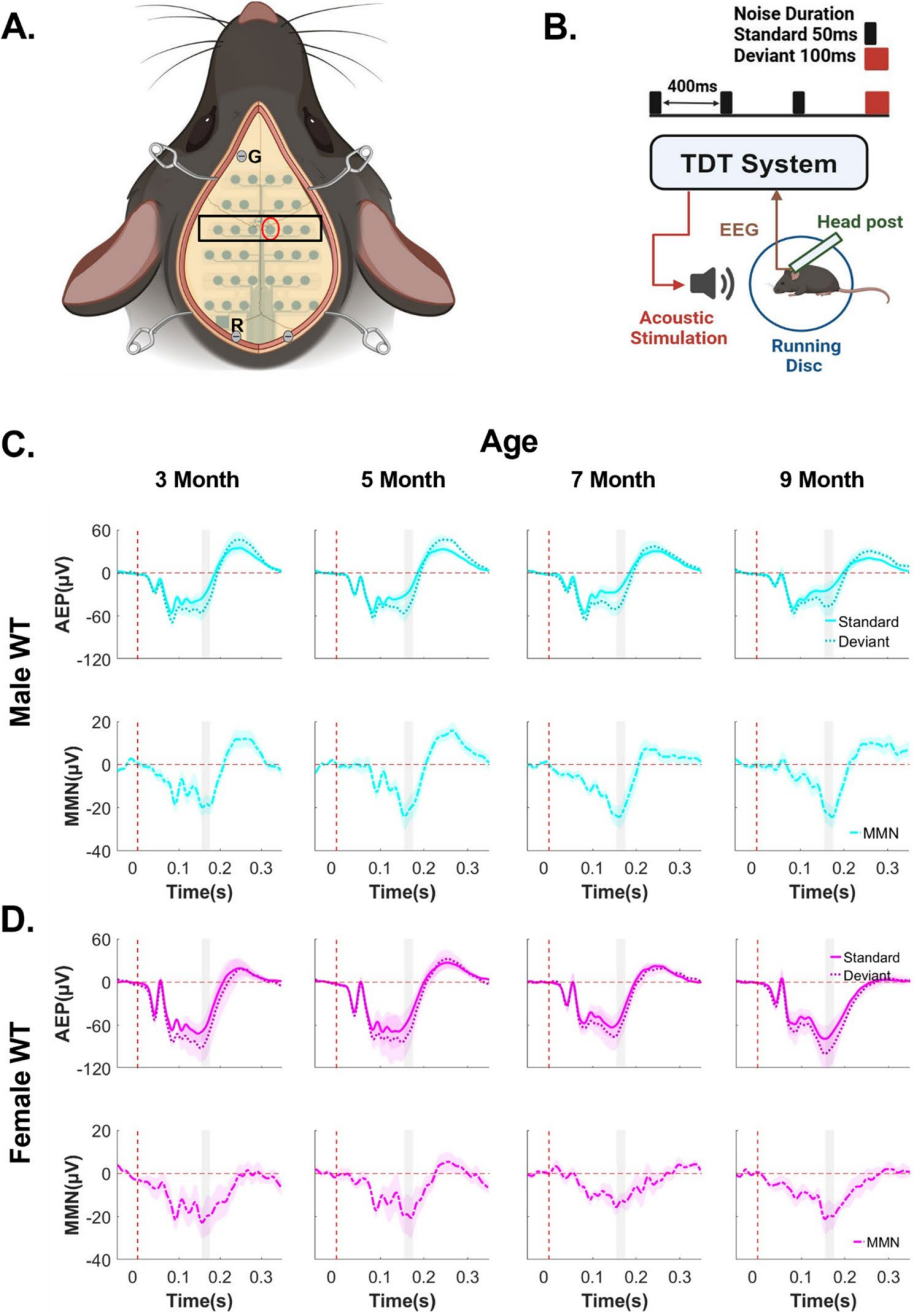
WT mice of both sexes showed robust auditory duration MMN responses. ***A***, Diagram of the 32- channel mouse EEG electrode array implanted on the skull of a mouse. The cross symbol on the array is positioned at the bregma. Screws are inserted for grounding (“G”), reference (“R”), and probe anchorage. Red circle shows the location of Ch21 (representative auditory duration MMN and AEP waveforms taken from this channel). Black square shows the location of all central electrodes (mean auditory duration MMN quantified from these channels). ***B***, Diagram shows the setup for recording mouse auditory duration MMN in mice. Animals are head-fixed to a head-post, with EEG and locomotion data recorded using a TDT system. A speaker is placed 10 cm in front of the animal. Standard tone: 50ms duration, 850 trials/session; Deviant tone: 100ms duration, 150 trials/session. ***C and D***, Trial and subject averaged AEP and auditory duration MMN waveforms for male (C) and female (D) WT mice aged 3 to 9 months, recorded from a centrally located channel (Ch21). The vertical dashed red line indicates stimulus onset. AEPs in response to standard tones (solid lines) and deviant tones (dotted lines) are presented, with the shaded areas representing the standard error of the mean (SEM). MMN (solid-dotted line), calculated as deviant AEP – standard AEP, are also presented with SEM indicated by shading. The gray rectangle indicates the MMN time window (160-180ms after stimulus onset). Male WT: *n* = 11 mice for 3-, 7- and 9- month-old groups; *n* = 14 for the 5-month-old group. Female WT: *n* = 6 mice for all ages. All animals were recorded longitudinally from 3 to 9 months of age, except three additional male mice recorded only at 5 months of age in pilot studies

EEG statistical analyses were performed and plotted using GraphPad Prism 10.2.3 (GraphPad Software, CA) and customized scripts in MATLAB. Mean auditory duration MMN from central electrodes (Fig. 1A) in the 160-180ms time window was calculated and analyzed using a repeated measures two-way ANOVA, followed by Tukey’s multiple comparisons for sex- and genotype-matched WT and *Cln3-/-* mice. Significance was defined as *p* < 0.05. Effect size was reported as partial eta-squared (η²_p_) [32–34]. A small effect has been defined as η² = 0.01, a medium effect as η² = 0.06, and a large effect as η² = 0.14 [35].

To explore the spatio-temporal dynamics of the data, two-sided unpaired *t* tests were performed between sex and age-matched WT and *Cln3-/-* mice across each electrode and time bin combination (electrode: Ch1-32, time: −50ms to 350ms in 20ms bins). The p-values were corrected for multiple comparisons by controlling the False Discovery Rate (FDR) [36] using MATLAB bioinformatics toolbox. Tests were computed for auditory duration MMN, AEP to standard stimuli, and AEP to deviant stimuli separately (i.e., WT MMN vs. *Cln3-/-* MMN, WT Standard AEP vs. *Cln3-/-* Standard AEP, WT Deviant AEP vs. *Cln3-/-* Deviant AEP). Results were considered significant if their FDR-corrected p-values were less than 0.05. T-statistics, instead of p-values, were plotted across all the electrodes and time bins using MATLAB to show the direction of changes. Significant results were plotted in dark red and blue, and non-significant results were plotted in light red and blue.

Furthermore, linear mixed effects (LME) models were fitted to the data in MATLAB to systematically characterize sex-, genotype- and age-dependent changes in auditory duration MMN, as well as standard and deviant AEPs (i.e., MMN, Standard AEP, or Deviant AEP ~ Age * Genotype * Sex + (1 | Subject)). Consistent with mean MMN quantification, standard and deviant AEPs in the LME models were averaged within the 160-180ms time window from central electrodes. Results were visualized as fitted values with 95% confidence intervals (CI), and F-tests for fixed-effects terms in the LME models were conducted to assess statistical significance.

For the statistical analysis of ABR data, two-way ANOVA analysis with Tukey’s multiple comparisons for sex-matched WT and *Cln3-/-* mice was performed to determine the effects of age and genotype on hearing thresholds. Significance was defined as *p* < 0.05.

## Results

### WT mice of both sexes showed robust auditory duration MMN responses

To determine whether auditory duration MMN can be reliably measured in WT mice, a 32-channel electrode array was implanted (Fig. 1A) in male and female WT mice, followed by the head-fixed recording of auditory neurophysiological responses using the duration MMN paradigm (Fig. 1B). Both male and female WT mice showed clear AEPs in response to standard and deviant stimuli across the skull with left-right symmetry (Supplementary Fig. 1). The auditory duration MMN in these mice showed a prominent anterior-to-central topographical distribution, with peak responses between 160-180ms post-stimulus onset across all electrodes (Supplementary Fig. 1). Due to the positioning of the reference and grounding electrodes (Fig. 1A), AEPs and auditory duration MMN responses were smaller at the most anterior and posterior electrodes (Supplementary Fig. 1).

Focusing on a centrally located electrode (Ch21), both male and female WT mice displayed robust auditory duration MMN responses from 3 to 9 months of age (Figs. 1C, D). In addition, topographical maps of AEPs for each electrode, averaged in 20ms increments, showed that AEPs in WT animals propagate from temporal regions near the Auditory Cortex (AC) to central parietal regions, and subsequently to frontal regions (Supplementary Fig. 1). This propagation pattern aligns with prior literature [11] and suggests that EEG recordings in the current study were not dominated by the volume conduction of electrical signals. Overall, WT mice of both sexes showed robust auditory duration MMN responses with minimal age-related changes in AEPs. These findings support the use of EEG recordings with the auditory duration MMN paradigm as a reliable approach for longitudinal studies.

### Cln3-/- mice showed sex-specific auditory duration MMN deficits

**Fig. 2:**
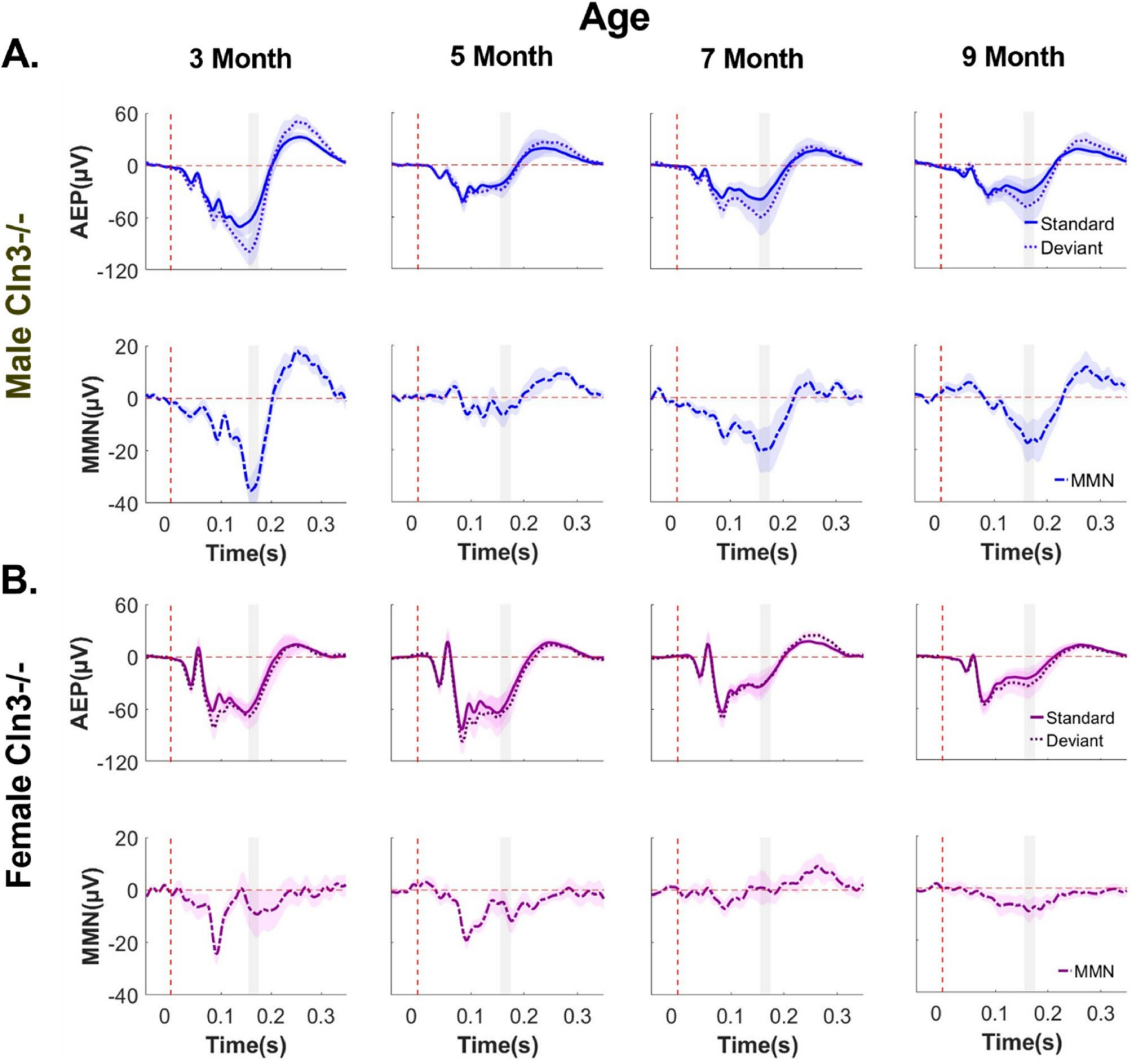
*Cln3-/-* mice showed sex-specific deficits in auditory duration MMN responses. ***A and B***, Trial and subject averaged AEP and auditory duration MMN waveforms for male (**A**) and female (**B**) *Cln3*-/- mice aged 3 to 9 months, recorded from Ch21. The vertical dashed red line indicates stimulus onset. AEPs in response to standard tones (solid lines) and deviant tones (dotted lines) are presented, with the shaded areas representing SEM. MMN (solid-dotted line), calculated as deviant AEP – standard AEP, are also presented with SEM indicated by shading. The gray rectangle indicates the MMN time window (160-180ms after stimulus onset). Male *Cln3-/-* mice showed a clear auditory duration MMN at 3 months of age, a diminished MMN at 5 months, and a subsequent recovery at 7 and 9 months (**A**), whereas female *Cln3-/-* mice showed diminished MMN across all ages (**B**). Male *Cln3-/-*: *n* = 8 mice for 3-, 7- and 9-month-old groups; *n* = 10 for the 5-month-old group. Female *Cln3-/-*: *n* = 7 mice for all ages. All animals were recorded longitudinally from 3 to 9 months of age, except two additional male mice recorded only at 5 months of age in pilot studies

Next, the effects of *Cln3-/-* genotype on auditory duration MMN responses were examined. As hypothesized, *Cln3-/-* mice showed an auditory duration MMN deficit (Fig. 2). Notably, there were sex-specific abnormalities in auditory duration MMN that are associated with disease progression in *Cln3-/-* mice.

In male *Cln3-/-* mice, auditory duration MMN responses displayed a dynamic trajectory. At 3 months of age, these mice exhibited strong negativity in the auditory duration MMN response (Fig. 2A). However, this response was nearly absent by 5 months of age (Fig. 2A). Surprisingly, auditory duration MMN recovered at 7 and 9 months of age (Fig. 2A), suggesting the involvement of potential compensatory neural mechanisms in these male mice.

In contrast, female *Cln3-/-* mice showed a trend of persistent auditory duration MMN deficits across all ages tested (3 to 9 months) (Fig. 2B). Reductions in the magnitude of the auditory duration MMN within the 160-180ms post stimulus time window, the period during which MMN is maximal in all WT mice, were evident at all ages (Fig. 2B). In summary, these findings reveal that *Cln3-/-* mice showed sex-specific auditory duration MMN abnormalities, in stark contrast to the consistent and robust responses observed in WT mice.

### MMN deficits emerged in younger Cln3-/- males were mitigated at older ages

**Fig. 3:**
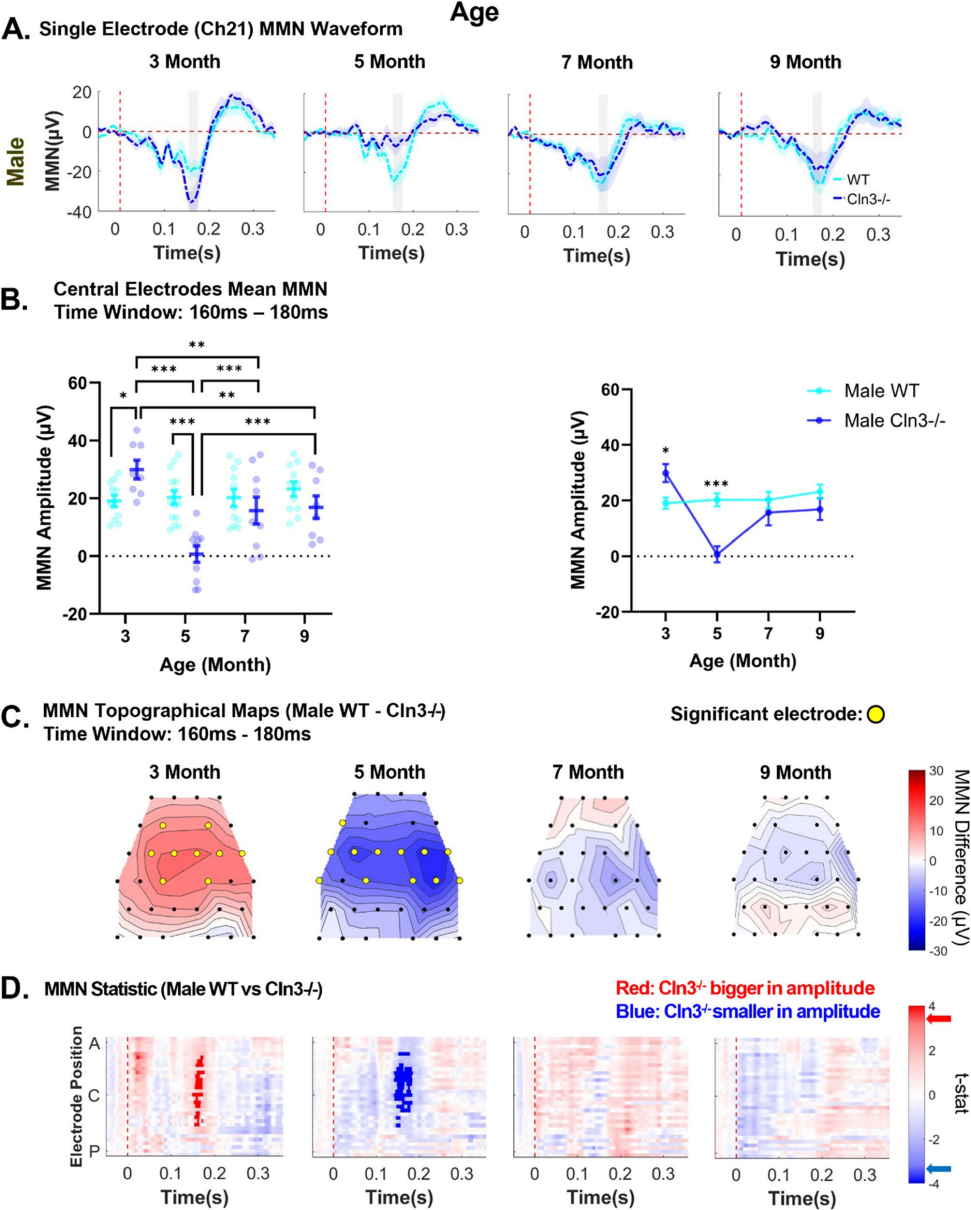
Male *Cln3*-/- mice showed late-age compensation of early-age MMN deficits. ***A***, trial- and subject-averaged auditory duration MMN waveforms for male WT mice (light blue) and male *Cln3-/-* mice (dark blue) at Ch21. Vertical dashed red line: stimulus onset. Gray rectangle: 160-180ms after stimulus onset. ***B***, Mean MMN amplitude within the 160-180ms time window at central electrodes for male WT and *Cln3-/-* mice. Individual data points for each animal and mean ± SEM. *Cln3-/-* mice showed significant differences in mean auditory duration MMN at 3 and 5 months of age compared to age-matched WT mice (3 month: *p* = 0.0181, 5 month: *p* < 0.0001). *Cln3-/-* mice also showed significant changes in mean auditory duration MMN across age (3 month vs. 5 month: *p* < 0.0001; 3 month vs. 7 month: *p* = 0.0015; 3 month vs. 9 month: *p* = 0.0026; 5 month vs. 7 month: *p* = 0.0003; 5 month vs. 9 month: *p* = 0.0003). ***C***, Topographical maps of the mean auditory duration MMN difference (WT MMN – *Cln3-/-* MMN). Red: *Cln3-/-* mice showed larger amplitudes; blue: *Cln3-/-* mice showed smaller amplitudes. Black dots: electrode positions; yellow dots: electrodes showing significant MMN differences. ***D***, Statistical differences in auditory duration MMN between male WT and *Cln3-/-* mice across all electrodes and the entire trial duration (32 electrodes × 400ms). Electrode positions: A (anterior), C (center) and P (posterior). Red: electrodes and time bins (in 20ms) where *Cln3-/-* mice showed larger amplitudes; blue: electrodes and time bins where *Cln3-/-* mice showed smaller amplitudes. Significant t-stat values: dark red and dark blue, based on the FDR-corrected p-value. Red and blue arrows on the color bar: significant t-stat value. Male WT: *n* = 11 for 3-, 7- and 9-month-old; *n* = 14 for the 5-month-old. Male *Cln3-/-*: *n* = 8 for 3-, 7- and 9-month-old; *n* = 10 for the 5-month-old

To further examine the effects of *Cln3* mutations on central auditory processing, the spatio-temporal patterns of auditory duration MMN responses in *Cln3-/-* mice were compared with those of sex- and age-matched WT mice. In male *Cln3-/-* mice, amplitudes of auditory duration MMN at a central electrode (Ch21) were elevated at 3 months of age, reduced at 5 months, and subsequently restored to approximately the WT level at 7 and 9 months (Fig. 3A). This age-dependent up-and-down modulation of auditory duration MMN magnitudes was consistently observed across electrodes at various spatial locations (Supplementary Fig. 2).

For statistical analysis, the mean auditory duration MMN was calculated by averaging responses within the 160-180ms time window from central electrodes (Fig. 1A), and the effects of age and genotype were tested (Fig. 3B). Repeated measures two-way ANOVA revealed a significant main effect of age (F(3,52) = 11.30, *p* < 0.0001, η²_p_ = 0.19 large effect size) and a significant genotype-by-age interaction (F(3,52) = 15.51, *p* < 0.0001, η²_p_ = 0.21, large effect size). The main effect of genotype was not significant (F(1,22) = 2.174, *P* = 0.1545, η²_p_ = 0.04, small effect size), likely due to the recovery of auditory duration MMN responses in male *Cln3-/-* mice at 7 and 9 months of age.

At 3 months of age, male *Cln3-/-* mice showed a significant increase in mean auditory duration MMN when compared to male WT mice (*p* = 0.0181, Tukey’s multiple comparisons test). Mean auditory duration MMN was then significantly decreased in 5 months old male *Cln3-/-* mice when compared to 5 months old male WT mice (*p* < 0.0001, Tukey’s multiple comparisons test). As male *Cln3-/-* mice aged to 7 and 9 months old, there was no age-matched genotype difference (7 month: *p* = 0.3695, 9 month: *p* = 0.1435, Tukey’s multiple comparisons test). Moreover, *Cln3-/-* mice showed significant differences in mean auditory duration MMN response across age (3 month vs. 5 month: *p* < 0.0001; 3 month vs. 7 month: *p* = 0.0015; 3 month vs. 9 month: *p* = 0.0026; 5 month vs. 7 month: *p* = 0.0003; 5 month vs. 9 month: *p* = 0.0003, Tukey’s multiple comparisons test), while male WT mice showed no age-related changes.

Next, the spatial distribution of MMN differences between WT and *Cln3-/-* male mice was examined using topographical maps. While male WT mice consistently showed robust auditory duration MMN across multiple electrodes and ages, male *Cln3-/-* mice displayed age-dependent variations in the spatial distribution of MMN (Supplementary Fig. 3). To better visualize genotype differences, topographical maps were generated by subtracting the MMN of male *Cln3-/-* mice (averaged within the 160-180ms time window) from that of male WT mice. At 3 months of age, male *Cln3-/-* mice showed greater MMN amplitudes compared to WT mice, with the most pronounced differences observed at central electrodes (Fig. 3C). By 5 months, the genotype difference reversed, with male *Cln3-/-* mice exhibiting reduced MMN amplitudes relative to WT mice, again showing the largest differences at central electrodes (Fig. 3C). At 7 and 9 months, little to no genotype difference in the spatial distribution of MMN was observed (Fig. 3C).

To test the statistical significance of genotype differences in the temporal and spatial distributions of MMN in male mice, pairwise *t*-tests were performed across all electrodes and time points and corrected for multiple comparisons by controlling the FDR (see Methods). At 3 months of age, male *Cln3-/-* mice showed a significant increase in MMN magnitude compared to WT mice at central electrodes, particularly within the 150–180ms post-stimulus time window (Fig. 3D). At 5 months, male *Cln3*-/- mice exhibited significantly reduced MMN magnitudes relative to WT mice, with the largest differences observed at central electrodes from 140–180ms post-stimulus onset (Fig. 3D). By 7 and 9 months of age, there were no significant differences in auditory duration MMN between male WT and *Cln3*-/- mice. In summary, while MMN magnitudes remained stable across age in male WT mice, male *Cln3-/-* mice showed age-dependent fluctuations in MMN amplitude, with an initial increase followed by a reduction and eventual restoration to levels comparable to WT mice at older ages.

### Persistent MMN deficits across age in Cln3-/- females

**Fig. 4:**
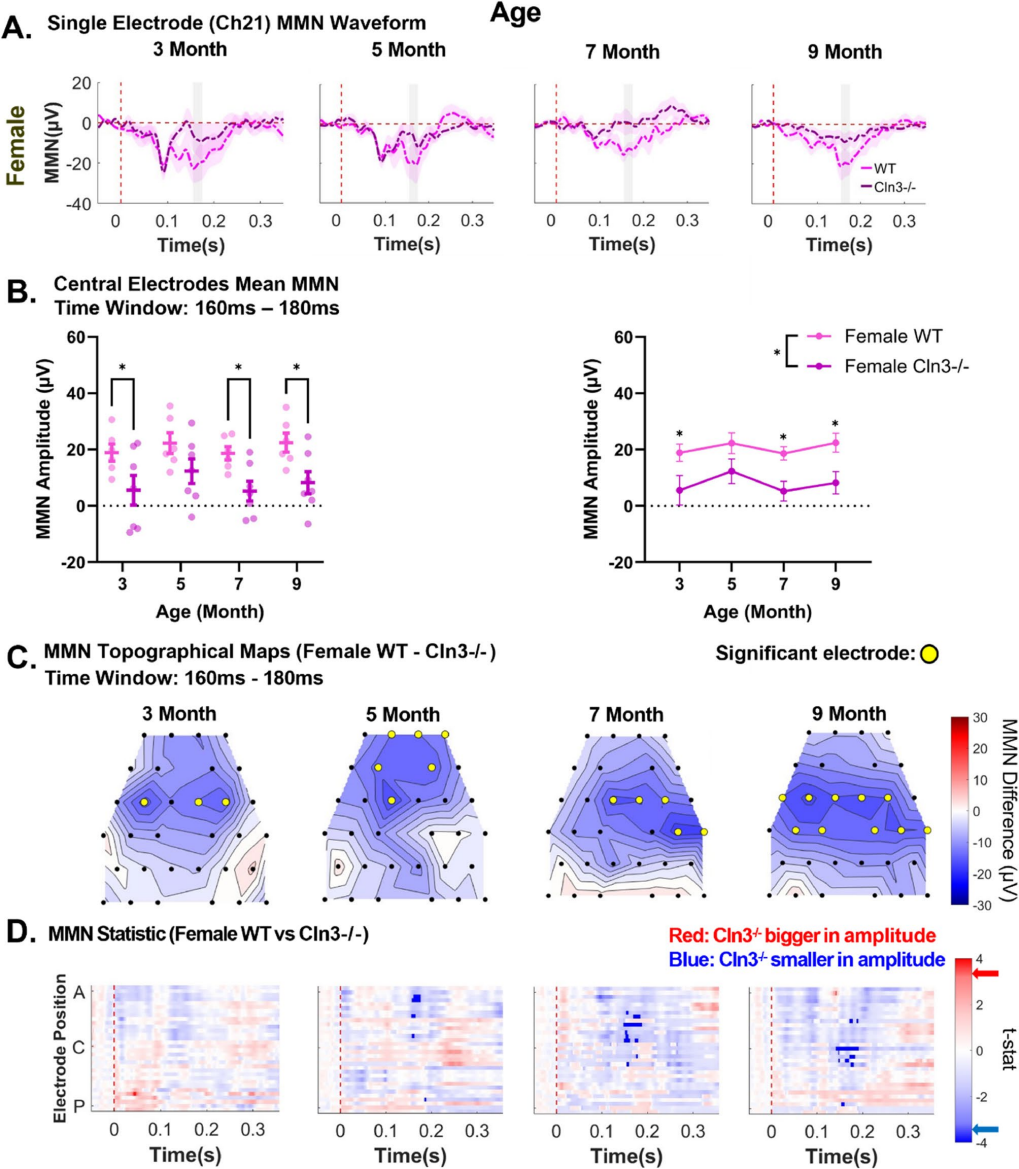
Female *Cln3*-/- mice showed persistent MMN deficits. ***A***, trial- and subject-averaged auditory duration MMN waveforms for female WT mice (light pink) and female *Cln3-/-* mice (dark pink) at Ch21. Vertical dashed red line: stimulus onset. Gray rectangle: 160-180ms after stimulus onset. ***B***, Mean MMN amplitude within the 160-180ms time window at central electrodes for female WT and *Cln3-/-* mice. Individual data points for each animal and mean ± SEM. Repeated measures two-way ANOVA showed a significant genotype main effect (*p* = 0.0232), with a reduction in the mean auditory duration MMN in female *Cln3-/-* mice compared to female WT mice. Age-matched genotype comparison showed that female *Cln3-/-* mice showed decline in mean auditory duration MMN at 3, 7 and 9 months of age (3 month: *p* = 0.02, 5 month: *p* = 0.0788, 7 month: *p* = 0.0194, 9 month: *p* = 0.0135). ***C***, Topographical maps of the mean auditory duration MMN difference between female WT and *Cln3-/-* mice (WT MMN – *Cln3-/-* MMN). Red: *Cln3-/-* mice showed larger amplitudes; blue: *Cln3-/-* mice showed smaller amplitudes. Black dots: electrode positions; yellow dots: electrodes showing significant MMN differences. ***D***, Statistical differences in auditory duration MMN between female WT and *Cln3-/-* mice across all electrodes and the entire trial duration (32 electrodes × 400ms). Electrode positions: A (anterior), C (center) and P (posterior). Red: electrodes and time bins (in 20ms) where *Cln3-/-* mice showed larger amplitudes; blue: electrodes and time bins where *Cln3-/-* mice showed smaller amplitudes. Significant *t*-stat values: dark red and dark blue, based on the FDR- corrected p-value. Red and blue arrows on the color bar: significant t-stat value. Female WT: *n* = 6 mice for all ages. Female *Cln3-/-*: *n* = 7 mice for all ages

Next, genotype differences in the spatio-temporal patterns of auditory duration MMN response in female mice were examined. The data showed that MMN magnitude in the 160-180ms post-stimulus time window was consistently reduced in *Cln3-/-* mice compared to WT mice across all ages at Ch21 (Fig. 4A). This reduction in MMN magnitude was also observed consistently across other electrodes (Supplementary Fig. 4).

Repeated measures two-way ANOVA analysis of central electrodes within the 160-180ms post-stimulus time window revealed a significant genotype effect, with female *Cln3-/-* mice showing reduced mean auditory duration MMN compared to female WT mice (F(1,11) = 6.947, *p* = 0.0232, η²_p_ = 0.3106, large effect size) (Fig. 4B). There were no significant effects of age or genotype-by-age interaction (Fig. 4B), as female *Cln3-/-* mice showed consistent auditory duration MMN deficits across age. For age-matched genotype comparisons, female *Cln3-/-* mice showed significantly decreased mean auditory duration MMN at 3, 7 and 9 months of age (3 month: *p* = 0.02, 5 month: *p* = 0.0788, 7 month: *p* = 0.0194, 9 month: *p* = 0.0135, Tukey’s multiple comparisons test).

Topographical maps of MMN genotype differences further confirmed that female *Cln3-/-* mice showed reduced MMN magnitudes compared to the WT in the 160-180ms time window across multiple electrodes and age groups (Fig. 4C). While female WT mice consistently showed robust auditory duration MMN across multiple electrodes and ages, female *Cln3-/-* mice displayed an overall lower magnitude in the spatial distribution of MMN (Supplementary Fig. 5). Detailed analyses of genotype differences at individual electrodes and time points also revealed significant MMN reductions in female *Cln3-/-* mice across the examined age groups (Fig. 4D). In summary, female *Cln3-/-* mice showed persistent auditory duration MMN deficits across ages.

### Cln3-/- and WT mice showed similar peripheral hearing from 3 to 9 months of age

**Fig. 5:**
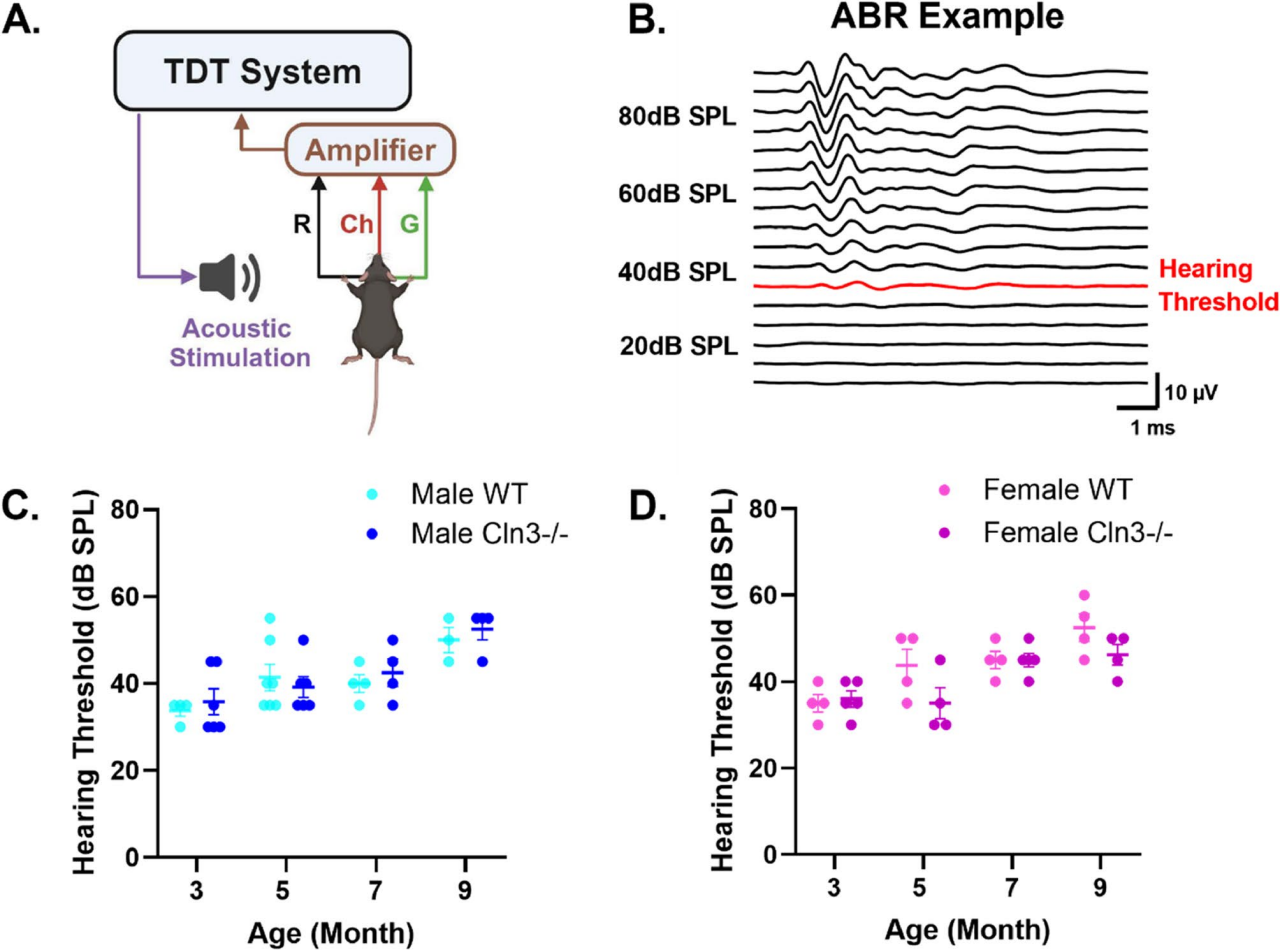
*Cln3-/-* and WT mice showed similar peripheral hearing from 3 to 9 months of age. ***A***, Diagram shows the click ABR recording set up. The TDT system is connected to a speaker for stimulus delivery. ABRs are recorded with three needle electrodes: black for reference (R), green for grounding (G), and red for active recording channel (Ch). The signals are amplified by an amplifier and processed by the TDT system. ***B***, The ABR waveforms of an example animal are shown, ranging from 10dB to 90dB. The hearing threshold for this animal is indicated in red. ***C***, Click ABR hearing thresholds for male WT (light blue) and male *Cln3-/-* mice (dark blue). Male *Cln3-/-* mice (*n* = 6 at 3-months, *n* = 6 at 5 months, *n* = 4 at 7 months, *n* = 4 for 9 months) showed no significant difference in click ABR hearing thresholds compared to age-matched male WT mice (*n* = 4 at 3 months, *n* = 7 at 5 months,*n* = 4 at 7 months,*n* = 3 at 9 months). ***D***, Click ABR hearing thresholds for female WT (light pink) and female *Cln3-/-* mice (dark pink). Female *Cln3-/-* mice (*n* = 5 at 3- and 7 months, *n* = 4 at 5 and 9 months) showed no significant difference in click ABR hearing thresholds compared to age-matched female WT mice (*n* = 4 for all ages). From 3 to 9 months of age, the hearing thresholds of all WT and *Cln3-/-* mice are below the testing sound level (i.e., 80dB SPL) used in the auditory duration MMN paradigm

To rule out the possibility that auditory duration MMN deficits originate from peripheral hearing loss, click ABR recording was performed to test hearing thresholds in WT and *Cln3-/-* mice of both sexes at the ages corresponding to the EEG recordings (Fig. 5A and B). Hearing thresholds in all animals were below the testing sound level (i.e., 80dB SPL) of our EEG stimuli (Fig. 5C and D). In male mice, there were no significant effects of genotype (F(1,30) = 0.3363, *p* = 0.5663, η²_p_ = 0.005, no effect, two-way ANOVA) or genotype-by-age interaction (F(3,30) = 0.3922, p-value = 0.7595, η²_p_ = 0.019, small effect size) (Fig. 5C). However, as expected, a significant age effect was observed (F(3,30) = 9.571, *p* = 0.0001, η²_p_ = 0.4725, large effect size), reflecting a gradual increase in hearing thresholds with age (Fig. 5C). Similarly, in female mice, there was a significant effect of age (F(3,26) = 11.17, *p* < 0.0001, η²_p_ = 0.4946, large effect size), but no significant effects of genotype (F(1,26) = 3.662, *p* = 0.0668, η²_p_ = 0.054, small effect size) or genotype-by-age interaction (F(3,26) = 1.681, *p* = 0.1955, η²_p_ = 0.074, medium effect size) (Fig. 5D).

The observed age-related differences in hearing thresholds for both sexes were consistent with the trend of age-related hearing loss (AHL) reported in the C57BL/6J mouse strain [37]. Previous studies have documented complete hearing loss in this strain between 12 and 15 months of age [38]. Additional recordings in 13-month-old mice confirmed that animals with complete hearing loss had no identifiable ABR or AEP waveforms (Supplementary Fig. 6). In conclusion, WT and *Cln3-/-* mice showed similar peripheral hearing thresholds at the ages corresponding to EEG recordings, indicating that auditory duration MMN deficits in *Cln3-/-* mice arise from central dysfunction rather than peripheral hearing loss.

### Male Cln3-/- mice showed age-dependent up-and-down modulation of AEPs

**Fig. 6:**
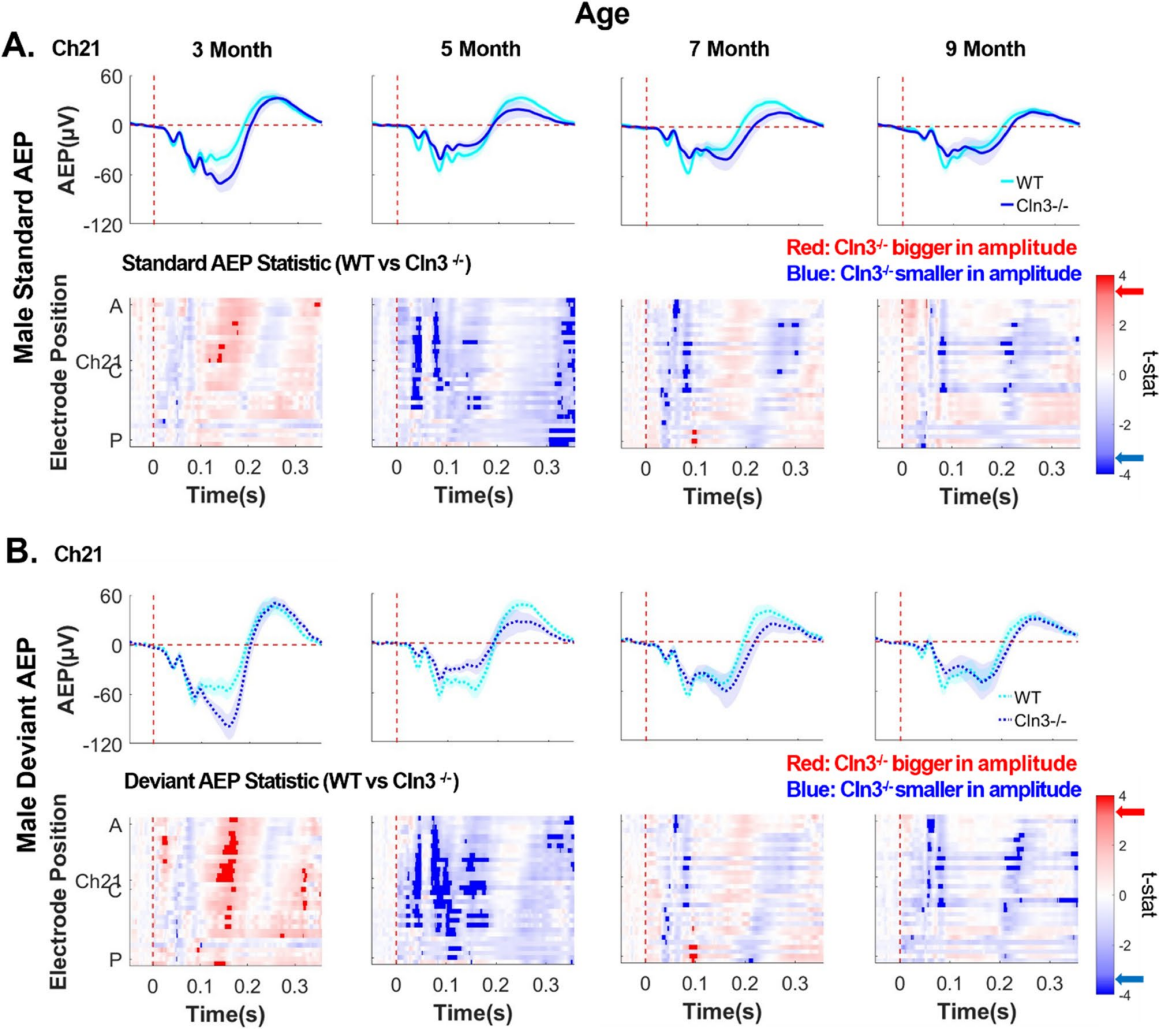
Male *Cln3*-/- mice showed late-age compensation of early-age AEP deficits. ***A***, top row, trial- and subject-averaged standard AEP waveforms for male WT mice (light blue) and male *Cln3-/-* mice (dark blue) at Ch21. The vertical dashed red line indicates stimulus onset. Bottom row, statistical differences in standard AEP between male WT and *Cln3-/-* mice were displayed across all electrodes and the entire trial duration (32 electrodes × 400ms). Results from *t*-tests were corrected for multiple comparisons using the FDR method. Significance was determined when the FDR-adjusted p-value was less than 0.05 (two-tailed). Electrode positions over the mouse skull are indicated from A (anterior) to C (center) and P (posterior). Red indicates electrodes and time bins (in 20ms) where *Cln3-/-* mice showed larger amplitudes (further away from zero compared to WT mice), while blue indicates electrodes and time bins where *Cln3-/-* mice showed smaller amplitudes (closer to zero compared to WT mice). Significant *t*-stat values are represented by dark red and dark blue, based on the FDR-corrected p-value. Red and blue arrows on the color bar indicate significant t-stat value. ***B***, Deviant AEP waveforms at Ch21 (top row) and *t*-stat values across all electrodes and the entire trial duration (bottom row). Male WT: *n* = 11 mice for 3-, 7- and 9-month-old groups; *n* = 14 for the 5-month-old group. Male*Cln3-/-*: *n* = 8 mice for 3-, 7- and 9-month-old groups; *n* = 10 for the 5-month- old group. Male *Cln3-/-* mice initially exhibited an enhanced auditory AEP response around the 160–180 ms MMN time window at 3 months of age, followed by a reduction at 5 months, and subsequently showed recovery at 7 and 9 months of age. Deviant AEP changes impacted more central electrodes and timepoints within the MMN window compared to standard AEPs

To investigate whether auditory duration MMN deficits in *Cln3-/-* mice originate from changes in auditory responses to standard stimuli, deviant stimuli, or both, AEPs between sex- and age-matched WT and *Cln3-/-* mice were compared. Standard AEPs in 3-month-old male *Cln3-/-* mice showed increased amplitude in several anterior-to-central electrodes around the typical MMN window compared to male WT mice (Fig. 6A). Deviant AEPs in these *Cln3-/-* mice also exhibited enhanced amplitude compared to WT mice, spanning more electrodes and time points than the standard AEPs (Fig. 6B). However, at 5 months of age, both standard and deviant AEPs in *Cln3-/-* mice were significantly reduced compared to WT mice, with reductions in deviant AEPs affecting more electrodes and time points (Fig. 6). By 7 and 9 months of age, both standard and deviant AEPs in male *Cln3-/-* mice returned to levels comparable to those of WT mice within the MMN time window (Fig. 6). Topographical maps of standard and deviant AEPs in male mice, averaged in 20ms time bins, further illustrate these spatiotemporal changes (Supplementary Fig. 7). In summary, male *Cln3-/-* mice showed age-dependent up-and-down modulation of AEP amplitudes, with more dramatic changes in deviant AEPs contributing to the genotype differences in MMN.

### Female Cln3-/- mice showed a progressive decline in AEPs

**Fig. 7:**
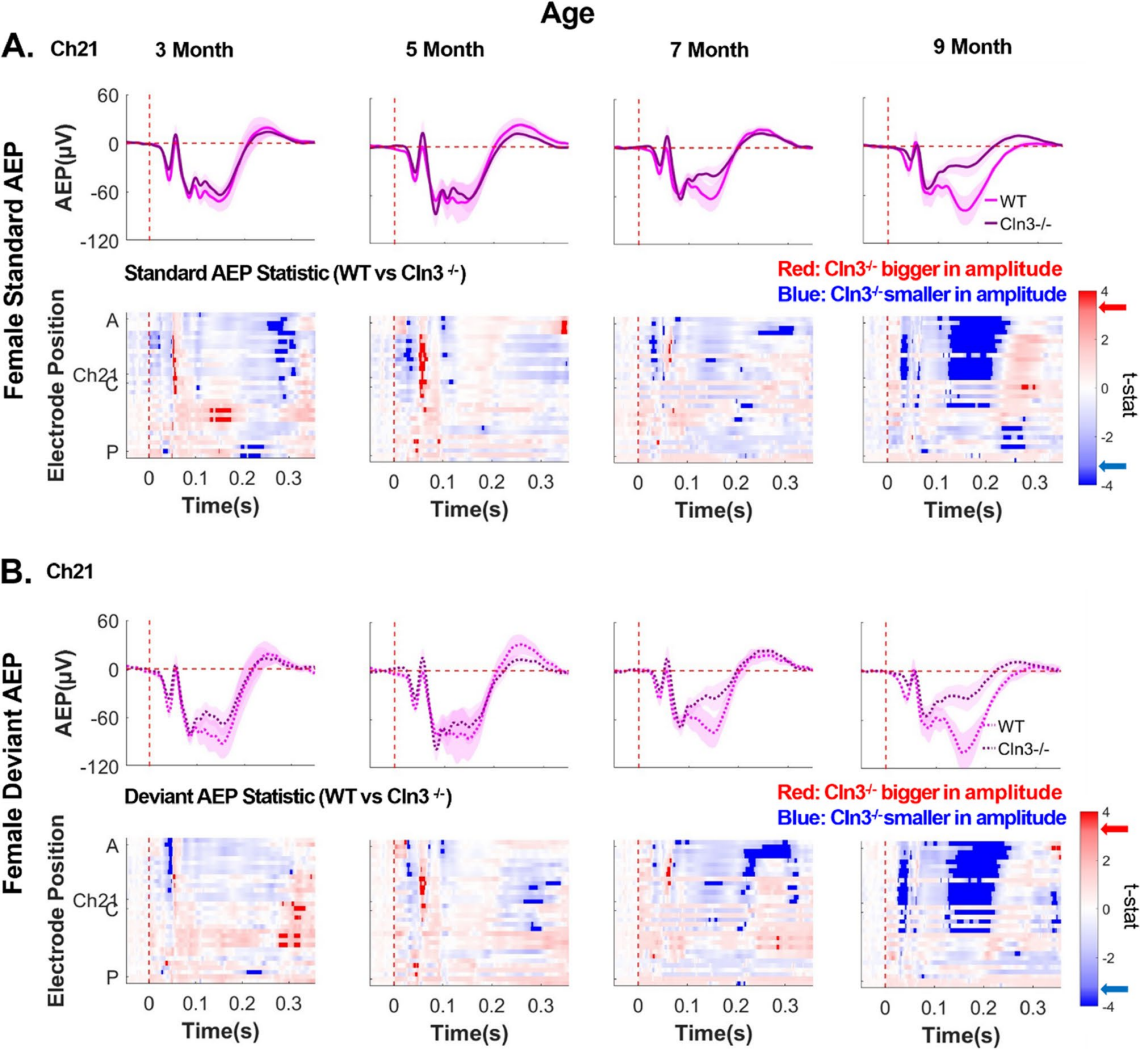
Female *Cln3-/- *mice showed a progressive decline in AEPs compared to WT mice. ***A***, top row, trial- and subject-averaged standard AEP waveforms for female WT mice (light pink) and *Cln3-/-* mice (dark pink) at Ch21. The vertical dashed red line indicates stimulus onset. Bottom row, statistical differences in standard AEP between female WT and *Cln3-/-* mice were displayed across all electrodes and the entire trial duration (32 electrodes × 400ms). Results from *t*-tests were corrected for multiple comparisons using the FDR method. Significance was determined when the FDR-adjusted p-value was less than 0.05 (two-tailed). Electrode positions over the mouse skull are indicated from A (anterior) to C (center) and P (posterior). Red indicates electrodes and time bins (in 20ms) where *Cln3-/-* mice showed larger amplitudes (further away from zero comparing to WT mice), while blue indicates electrodes and time bins where *Cln3-/-* mice showed smaller amplitudes (closer to zero comparing to WT mice). Significant *t*-stat values are represented by dark red and dark blue, based on the FDR-corrected p-value. Red and blue arrows on the color bar indicate significant t-stat value. ***B***, Deviant AEP waveforms at Ch21 (top row) and *t*-stat values across all electrodes and the entire trial duration (bottom row). Female WT: *n* = 6 mice for all ages. Female *Cln3-/-*: *n* = 7 mice for all ages. AEPs around the typical MMN time window (160–180ms) progressively decreased with age in female *Cln3-/-* mice. By 9 months of age, female *Cln3-/-* mice showed significantly reduced standard and deviant AEPs at anterior to central electrodes

Age-related changes in AEPs in female mice were also examined. While female WT mice maintained consistent AEPs from 3 to 9 months of age, female *Cln3-/-* mice showed a gradual decline in AEP magnitudes (Fig. 7). Both standard and deviant AEPs around the typical MMN time window were reduced in female *Cln3-/-* mice compared to age-matched WT mice, with the most pronounced differences observed at 9 months of age in anterior-to-central electrodes (Fig. 7A, B). Topographical maps of standard and deviant AEPs, averaged in 20ms time bins, further illustrate these spatiotemporal changes in female mice (Supplementary Fig. 8). In summary, female *Cln3-/-* mice demonstrated a progressive decline in both standard and deviant AEPs, contributing to a persistent trend of auditory duration MMN deficits.

### Sex-specific and age-dependent alterations of auditory duration MMN and AEPs in Cln3-/- mice

To summarize the alterations in auditory duration MMN and AEPs in *Cln3-/-* and WT mice, linear mixed-effects (LME) models were applied to the data (see Methods). Overall, significant three-way Genotype-by-Age-by-Sex interactions were observed for MMN and AEPs (MMN: *p* = 5.65e-06; Standard AEP: *p* = 0.0346; Deviant AEP: *p* = 1.05e-6; F-tests for fixed-effects terms in LME).

**Fig. 8:**
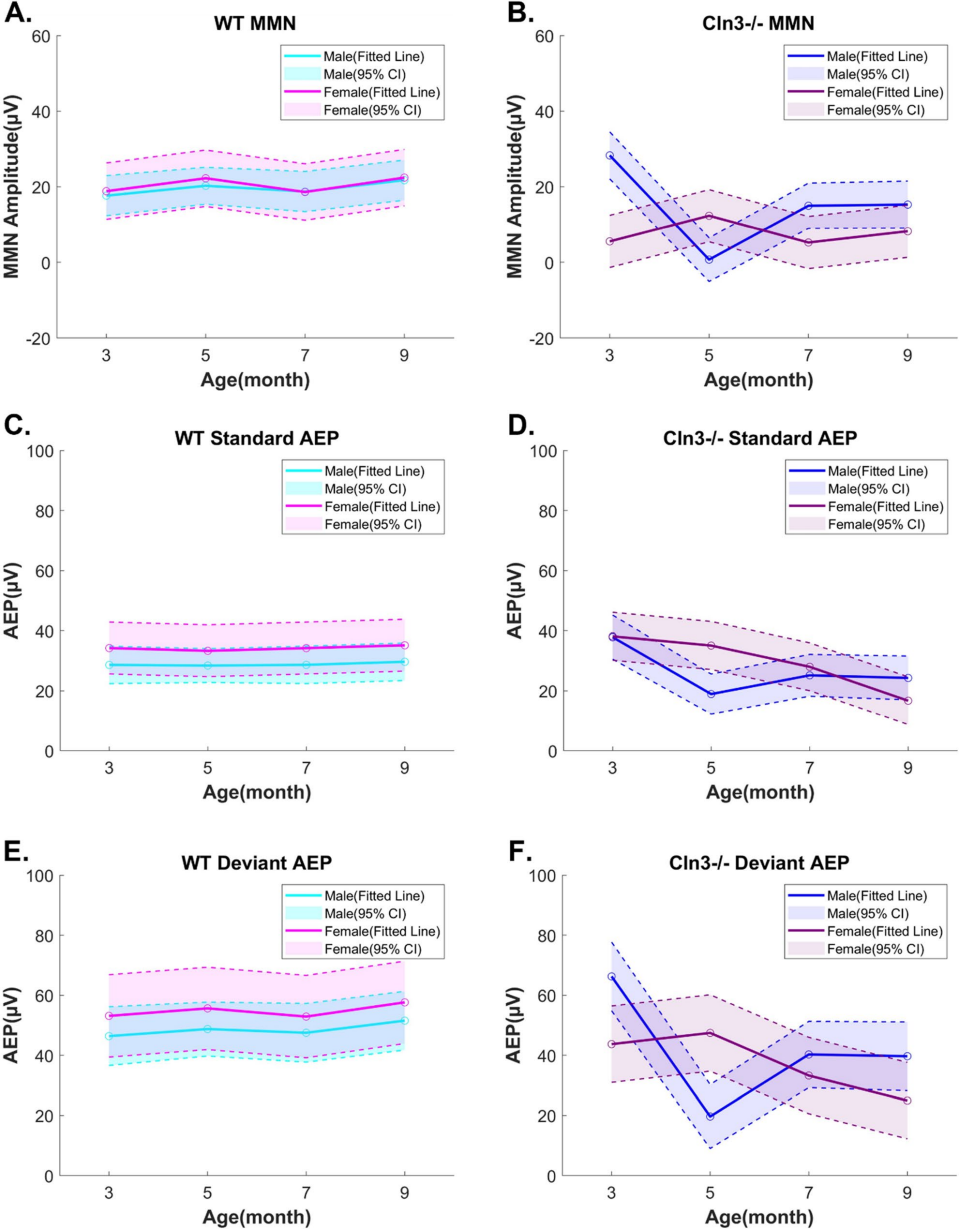
Sex-specific and age-dependent alterations of auditory duration MMN and AEPs in *Cln3-/-* mice. Linear mixed effects (LME) models were applied to mean auditory duration MMN, standard AEP, and deviant AEP data in genotype-matched mice of both sexes across ages. MMN, Standard AEP, or Deviant AEP ~ Age * Genotype * Sex + (1 | Subject). F-tests for fixed-effects terms in the LME models revealed significant three-way Genotype-by-Age-by-Sex interactions (MMN: *p* = 5.65e-06; Standard AEP: *p* = 0.0346; Deviant AEP: *p* = 1.05e-6). MMN and AEP data were averaged from 160-180ms post stimulus onset at central electrodes. Circle: mean fitted values for each group at different age points. Solid line: connecting fitted values for each group. Dash line and shade: 95% confidence interval (CI). Male WT: light blue. Female WT: light pink. *Male Cln3-/-*: Dark blue. Female *Cln3-/-*:Dark pink. ***A***, WT mice auditory duration MMN LME model. ***B***, *Cln3-/-* mice auditory duration MMN LME model. ***C***, WT mice standard AEP LME model. ***D***, *Cln3-/-* mice standard AEP LME model. ***E***, WT mice deviant AEP LME model. ***F***, *Cln3-/-* mice deviant AEP LME model. WT mice showed consistent AEPs with robust MMN across age. There was no sex difference between WT mice. *Cln3-/-* mice showed sex-specific and age-dependent alterations of auditory duration MMN and AEPs

WT mice showed consistent AEPs with robust auditory duration MMN responses across ages (Fig. 8A, C, E). No statistically significant sex differences were observed between male and female WT mice, because the fitted values for each group consistently fell within the 95% confidence interval of the other (Fig. 8A, C, E).

In male *Cln3-/-* mice, auditory duration MMN and AEPs followed a non-linear trajectory, characterized by an elevated response at 3 months, a decline at 5 months, and partial recovery at 7 and 9 months (Fig. 8B, D, F). In contrast, female *Cln3-/-* mice exhibited persistently reduced MMN responses relative to WT across the same age range, accompanied by a progressive, age-dependent decline in AEPs.

Sex-specific differences were most evident at several key time points. At 3 months, male *Cln3-/-* mice showed significantly greater MMN responses and higher deviant AEPs—but not standard AEPs—compared to females (Fig. 8B, D, F). This was followed by a marked decline in MMN at 5 months, at which point males also showed significantly lower standard and deviant AEPs compared to female *Cln3*-/- mice. By 7 months, MMN responses and AEPs in males rebounded to levels comparable to age-matched females. By 9 months, deviant AEPs in female *Cln3-/-* mice had further declined to levels significantly lower than those observed in males.

In summary, while WT mice of both sexes exhibited consistent AEPs and robust auditory duration MMN responses from 3 to 9 months of age, *Cln3-/-* mice demonstrated clear sex- and age-dependent alterations in both MMN and AEPs. In males, MMN responses fluctuated non-linearly with age, reflecting variable trajectories in standard and deviant AEPs. In contrast, females displayed a progressive, parallel decline in both standard and deviant AEPs, resulting in persistently reduced MMN responses across age. Combined with evidence of intact peripheral hearing thresholds, these results suggest age- and sex-dependent central auditory processing deficits in *Cln3-/-* mice.

## Discussion

This study presents the first evidence of age-related and sex-specific central auditory processing abnormalities in the *Cln3-/-* mouse model of Batten disease. These findings highlight the importance of systematically evaluating both sexes across multiple ages to fully understand the physiological consequences of *Cln3* mutations and to guide the development of targeted disease management and treatment strategies.

Findings of the current study validate the utility of the auditory duration MMN paradigm in murine models as a reliable tool for longitudinal assessment of sensory processing deficits. While MMN paradigms have been used in some rodent models of brain disorders, for example, to capture deficits potentially related to schizophrenia [39, 40], their use for long-term tracking of neurophysiological deficits remains limited. This study demonstrates that auditory neurophysiological responses in WT mice are consistent across six months of adult life, confirming the reliability of the auditory duration MMN paradigm for assessing auditory sensory memory and deviance detection over time [41].

The observed alterations in auditory neurophysiological responses in *Cln3-/-* mice likely stem from sex-specific patterns of disease progression, as WT mice of both sexes showed minimal age-related changes in auditory duration MMN and AEPs across the studied age range (3–9 months) studied. A promising avenue for further investigation is the relationship between ceroid lipofuscin accumulation, which is a pathological hallmark of CLN3 disease [42], and auditory neurophysiological deficits. Ceroid lipofuscin storage material is known to contain substantial amounts of subunit C of mitochondrial ATP synthase (SCMAS) [17], making SCMAS a widely used histopathological marker for CLN3 disease [43]. While previous research did not specifically examine auditory brain regions for SCMAS expression in *Cln3* mouse models, one recent study suggested that male *Cln3* mutant mice accumulate significantly more SCMAS in visual cortex, somatosensory cortex, thalamus, and hippocampus at postnatal day 70, compared to female *Cln3* mutant mice [29]. These findings underscore the importance of accounting for sex differences when investigating the relationship between neuropathological features of CLN3 disease and associated neurophysiological impairments.

The mechanisms underlying late-age compensation of auditory deficits in male *Cln3-/-* mice, but not in females, present an intriguing direction for future investigation. Investigating neuropathological markers may offer key insights into these sex-specific differences in compensatory capacity. Most prior studies of *Cln3* mouse models have focused on middle-aged to older animals (6–24 months), where pathological features such as storage body accumulation [19] and glial activation [28] become more pronounced. However, sex differences in storage material accumulation at older ages remain largely unexplored in *Cln3* mouse models [27]. Notably, studies in the *Cln6* model of Batten disease revealed that male mutant mice accumulate more storage material in the somatosensory cortex and thalamus at 2 months of age, whereas female *Cln6* mutant mice surpass males in storage material by 6 months of age in these same regions [44]. A similar progression in *Cln3* mutant females could potentially explain the irreversible auditory dysfunctions observed at later ages in this study.

However, previous studies also suggested that the accumulation of ceroid lipofuscin alone does not necessarily correlate with neural or behavioral deficits [17, 45]. Further investigation into the neuropathology and pathophysiology in *Cln3-/-* mice along auditory processing pathways may help pinpoint specific regions and cell types involved in central auditory processing deficits and the observed compensatory responses in males. Importantly, the identification of potential compensatory processes in male *Cln3-/-* mice in the current study introduces a novel avenue for therapeutic discovery aimed at preserving or restoring auditory function in Batten disease.

Of note, multiple *Cln3* mouse models have been developed, each carrying distinct mutations that produce varying phenotypic profiles. The *Cln3*-/- mouse model used in this study was generated by deleting exons 1–6 of the *Cln3* gene, resulting in a complete loss-of-function allele and absence of CLN3 protein expression [17]. In contrast, the *Cln3*Δex7/8 knock-in model deletes exons 7 and 8 to mimic a common human mutation [45]; it retains expression of a truncated transcript, but the resulting protein is dysfunctional. More recently, the *Cln3*Q352X model was created by introducing a nonsense point mutation (Q352X) that replicates a less common human variant and leads to premature truncation of the CLN3 protein [28]. These models differ in the timing and severity of pathological and behavioral deficits: the *Cln3*-/- model typically displays earlier and more pronounced accumulation of storage material, neuroinflammation, and behavioral abnormalities, making it well suited for time-sensitive biomarker validation and therapeutic screening [18–20], whereas the *Cln3*Δex7/8 and *Cln3*Q352X models are well suited for studying molecular pathogenic mechanisms and mutation-specific gene therapies [28, 46, 47].

Although *Cln3* mouse models share broad phenotypic features, patterns of disease progression can vary depending on the model. Observed differences often depend on the specific outcome measured, as well as variables such as sex and genetic background. While each model has its strengths, it remains unclear whether the auditory MMN and AEP phenotypes identified in this study are conserved across models. Future cross-model comparisons using the EEG-MMN paradigm will be crucial to assess the generalizability of central auditory processing deficits across different *Cln3* mutations.

Our study helps bridge a critical gap between pre-clinical and clinical research in CLN3 disease. Widely used assays in *Cln3* mouse models, such as invasive electrophysiological recordings [25] and behavioral assessments, ranging from maze tasks [48] to open field [29] and rotarod tests [47], are not easily translatable to human studies. The MMN-based EEG paradigm provides a noninvasive neurophysiological measure of brain function that is readily comparable across species and complements established ocular assessments in *Cln3* mouse research. For example, electroretinograms (ERGs) have shown reduced a- and b-wave amplitudes, as well as decreased b/a-wave ratios, in homozygous *Cln3*Δex7/8 mice at 12 months of age, suggesting inner retinal degeneration [49]. Incorporating both EEG and ERG assessments could provide a more comprehensive evaluation of cortical and retinal dysfunction in CLN3 disease.

Importantly, our previous work identified auditory duration MMN deficits in individuals with CLN3 disease [9], and the current study demonstrates highly consistent abnormalities in *Cln3-/-* mice. This validates auditory duration MMN as a translational neurophysiological biomarker, enabling cross-species evaluation and comparison of sex- and age-specific progression in pathophysiological phenotypes. For instance, the drastic reductions of auditory duration MMN and AEPs in old female *Cln3-/-* mice has important clinical relevance, as female patients with CLN3 disease experience rapid progression towards severe symptoms, despite later onset compared to males [7, 26, 50]. Due to the low prevalence of CLN3 disease (~ 1:100,000) in the general population [2], there is limited availability of longitudinal neurophysiological data in human patients. While some clinical studies have reported sex-related differences in disease severity and progression [7, 26], the presence of sex-specific and age-related changes in auditory MMN and AEPs has not yet been systematically investigated. Larger, longitudinal EEG studies in CLN3 patient cohorts will be needed to determine whether the patterns observed in the mouse model extend to the human disease trajectory.

Auditory duration MMN offers a sensitive and scalable neurophysiological measure for evaluating treatment efficacy in CLN3 disease, particularly in early-stage clinical trials where traditional functional assessments may lack sensitivity. Several therapeutic strategies that have shown promise in animal models have not yet yielded significant clinical benefits in humans, highlighting the need for more translatable outcome measures. For example, mycophenolate, an immunosuppressive agent, improved motor coordination and reduced neuroinflammation in *Cln3-/-* mice following daily oral administration beginning at postnatal day 32 for up to 150 days [51]. However, in a randomized, double-blind, placebo-controlled crossover trial in CLN3 patients, two 8-week treatment periods (separated by a 4-week washout) showed no significant improvement in motor function or behavior as assessed by the Unified Batten Disease Rating Scale (UBDRS) [52]. A similar discrepancy was observed with trehalose, a disaccharide that enhances autophagy [53]. In *Cln3*Δex7/8 mice, trehalose reduced lysosomal storage accumulation and neuroinflammation [54], yet a 12-month clinical trial (6 months of treatment followed by a 6-month washout) did not produce significant changes on the UBDRS in CLN3 patients [55]. Taken together, these findings underscore the challenge of translating preclinical success into clinical efficacy, particularly when outcome measures rely solely on overt behavioral scales. Integrating MMN-based EEG measures into future preclinical and clinical trials could offer an important functional readout of cortical integrity and sensory processing, enabling detection of treatment effects that may otherwise go unnoticed.

MMN-based EEG paradigms are well suited for evaluating the efficacy of emerging therapies including pharmacological agents and gene therapies. For example, AAV9-mediated delivery of human *CLN3* has been shown to restore protein expression [56] and reduce ceroid lipofuscin accumulation in the hippocampus and somatosensory cortex of *Cln3*Δex7/8 mice [57]. Miglustat (Batten-1), an enzyme inhibitor that limits glucosylceramide accumulation [58], has shown efficacy in other lysosomal storage disorders such as Niemann-Pick disease type C and type 1 Gaucher disease [59, 60]. An open-label Phase I/II trial in CLN3 disease (NCT05174039) is currently underway, and preliminary findings suggest that miglustat treatment may help stabilize visual acuity and slow motor symptom progression. The auditory duration MMN paradigm provides a translational and objective method to assess the functional impact of such interventions, making it a valuable addition to future therapeutic trials.

Auditory duration MMN provides a functional, neurophysiological measure that is complementary to existing biochemical biomarkers for CLN3 disease. Glycerophosphodiesters (GPDs) have emerged as a promising class of biomarkers, reflecting disrupted cellular metabolism and lysosomal function in CLN3 disease [61, 62]. GPDs are lipid degradation intermediates normally cleared up by functional lysosomes [63]. In CLN3 disease, impaired lysosomal activity leads to the accumulation of GPDs in brain tissue, cerebrospinal fluid (CSF), and plasma across multiple model systems, including *Cln3* mouse models, the *Cln3*Δex7/8 minipig model, and CLN3-affected individuals [64–66]. Whereas GPDs reflect underlying cellular and lysosomal dysfunction, EEG-based auditory duration MMN captures higher-order, systems-level alterations in central sensory processing. Combining GPDs with MMN-based measures may offer a more comprehensive and translational framework for early diagnosis, disease monitoring, and therapeutic evaluation in CLN3 disease, as it links biochemical disruptions with functional brain outcomes.

EEG recordings with the auditory duration MMN paradigm are appliable to other neurodevelopmental disorders. Previous work in Rett Syndrome [67], 22q11.2 deletion syndrome [68], and Cystinosis [69, 70] has identified MMN deficits in human participants. Expanding MMN studies to murine models of these disorders could facilitate longitudinal tracking of disease progression and exploration of sex and age differences. Validation of MMN as a translational neurophysiological biomarker across disorders may also aid in establishing common endophenotypes for various neurodevelopmental conditions.

A limitation of the current study is related to the spatial resolution of EEG. Given that EEG is a measure of synchronized electrical activity from large neuronal populations [71], it is difficult to pinpoint the exact brain location underlying auditory duration MMN and AEP differences in WT and *Cln3-/-* mice. Since MMN is a higher-order brain response generated from interactions between auditory thalamocortical circuits and frontal-temporal brain regions [72], further investigations using complementary approaches are needed to elucidate the neural mechanisms driving these deficits.

## Conclusions

In conclusion, WT mice of both sexes maintained normal central auditory processing over time, whereas male *Cln3-/-* mice showed restoration of early deficits and female *Cln3-/-* mice exhibited a progressive decline. These findings provide a foundation to characterize sex- and disease progression-specific sensory processing deficits, uncover underlying neural mechanisms, and utilize auditory duration MMN as a cross-species neurophysiological biomarker. 

## Supplementary Information

Supplementary Material 1.

## Data Availability

The data used in the current study will be available upon reasonable request.

## Abbreviations

NCL: Neuronal Ceroid Lipofuscinosis
LSD: Lysosomal Storage Disorder
WT: Wild-Type
CLN3: Ceroid Lipofuscinosis Neuronal 3
EEG: Electroencephalography
AEP: Auditory Evoked Potential
MMN: Mismatch Negativity
ABR: Auditory Brainstem Response
FDR: False Discovery Rate
LME: Linear Mixed Effects
CI: Confidence Interval
