# Supplementary material for: Sex-specific and age-related progression of auditory neurophysiological deficits in the Cln3 mouse model of Batten disease
Source: J Neurodev Disord. 2025 Nov 6;17:67. doi: 10.1186/s11689-025-09652-2 (PMC12590631; doi:10.1186/s11689-025-09652-2)
Supplement: Supplementary file 1 — Supplementary Material 1. [file 11689_2025_9652_MOESM1_ESM.docx]

**Supplementary Material for:**

**Sex-specific and age-related progression of auditory neurophysiological deficits in the *Cln3* mouse model of Batten disease**

**Authors:** Yanya Ding*, Jingyu Feng*, Viollandi Prifti, Grace A. Rico, Alexander G. Solorzano, Hayley E. Chang, Edward G. Freedman, John J. Foxe**^#^**, Kuan Hong Wang**^#^**

**Affiliation:** Department of Neuroscience, University of Rochester Medical Center, Rochester, NY, 14642, USA

*** First authors**

**^#^ Corresponding authors:**

John J. Foxe: [john_foxe@urmc.rochester.edu](mailto:john_foxe@urmc.rochester.edu)

Kuan Hong Wang: [kuanhong_wang@urmc.rochester.edu](mailto:kuanhong_wang@urmc.rochester.edu)


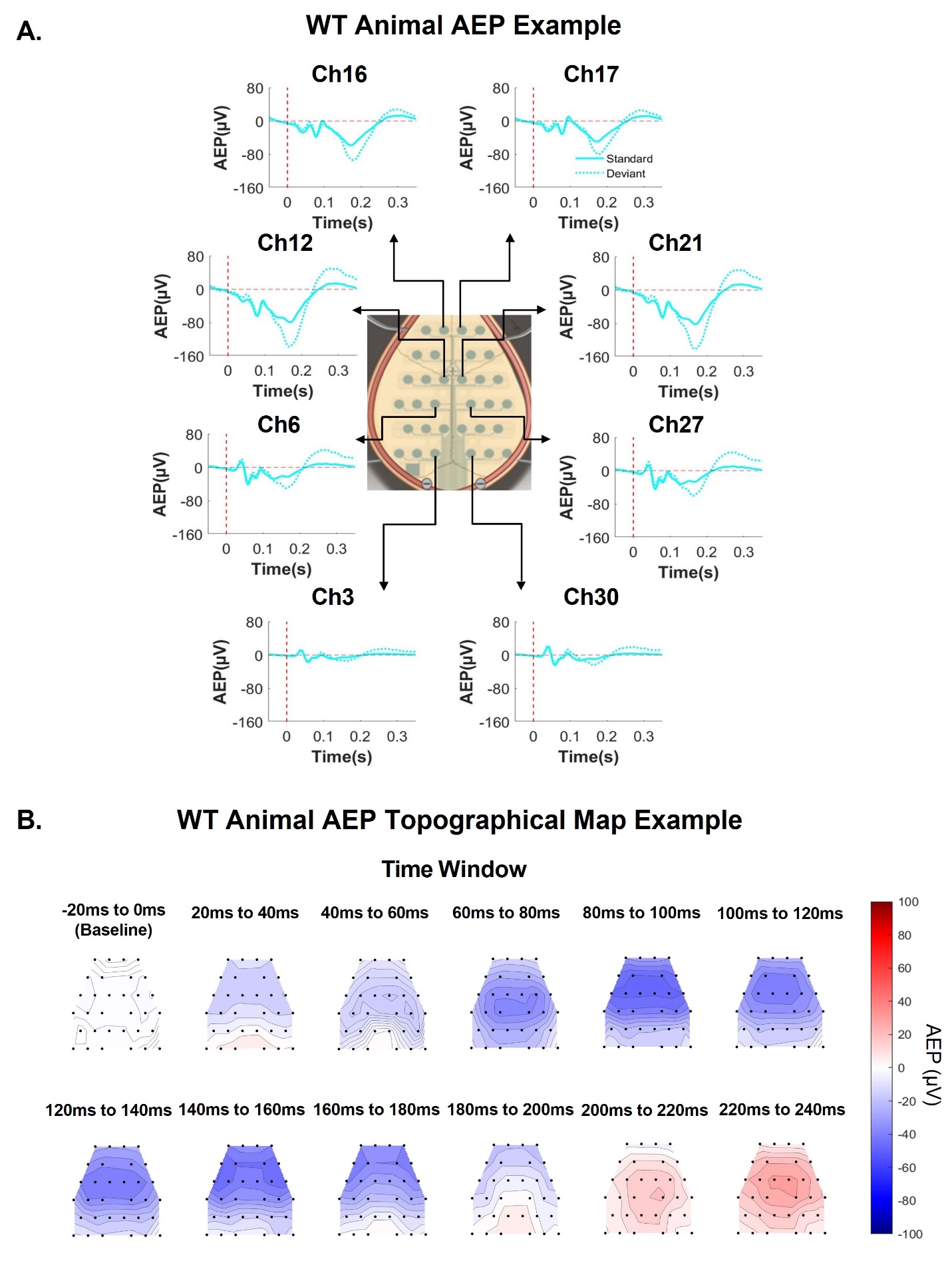


**Supplementary Figure 1. EEG mice showed robust responses to both standard and deviant stimuli in spatial distribution. *A,*** Standard and deviant AEP waveforms from one representative WT male mouse. Ch stands for channel number. The vertical dash red line indicates the onset of stimuli, and the horizontal dash red line indicates 0. Averaged AEP to standard tones is presented with solid cyan line and averaged AEP to deviant tones is presented with dotted cyan line. The example mouse showed clear AEPs to both standard and deviant tones from anterior to posterior region over the scalp, with MMN seen maximal at the center channels (i.e., Ch12 and Ch21). AEPs have left-right hemisphere symmetry. AEPs at most posterior channels (i.e., Ch3 and Ch30) are smaller due to referencing. ***B,*** Topographical maps of standard AEP in a 20ms time window from one representative WT male mouse. Black dots indicate the position of EEG electrodes. The color bar shows the positivity (red) and negativity (blue) of the AEP response. AEPs in WT animals propagate from temporal regions near the Auditory Cortex (AC) to central parietal regions, and subsequently to frontal regions.


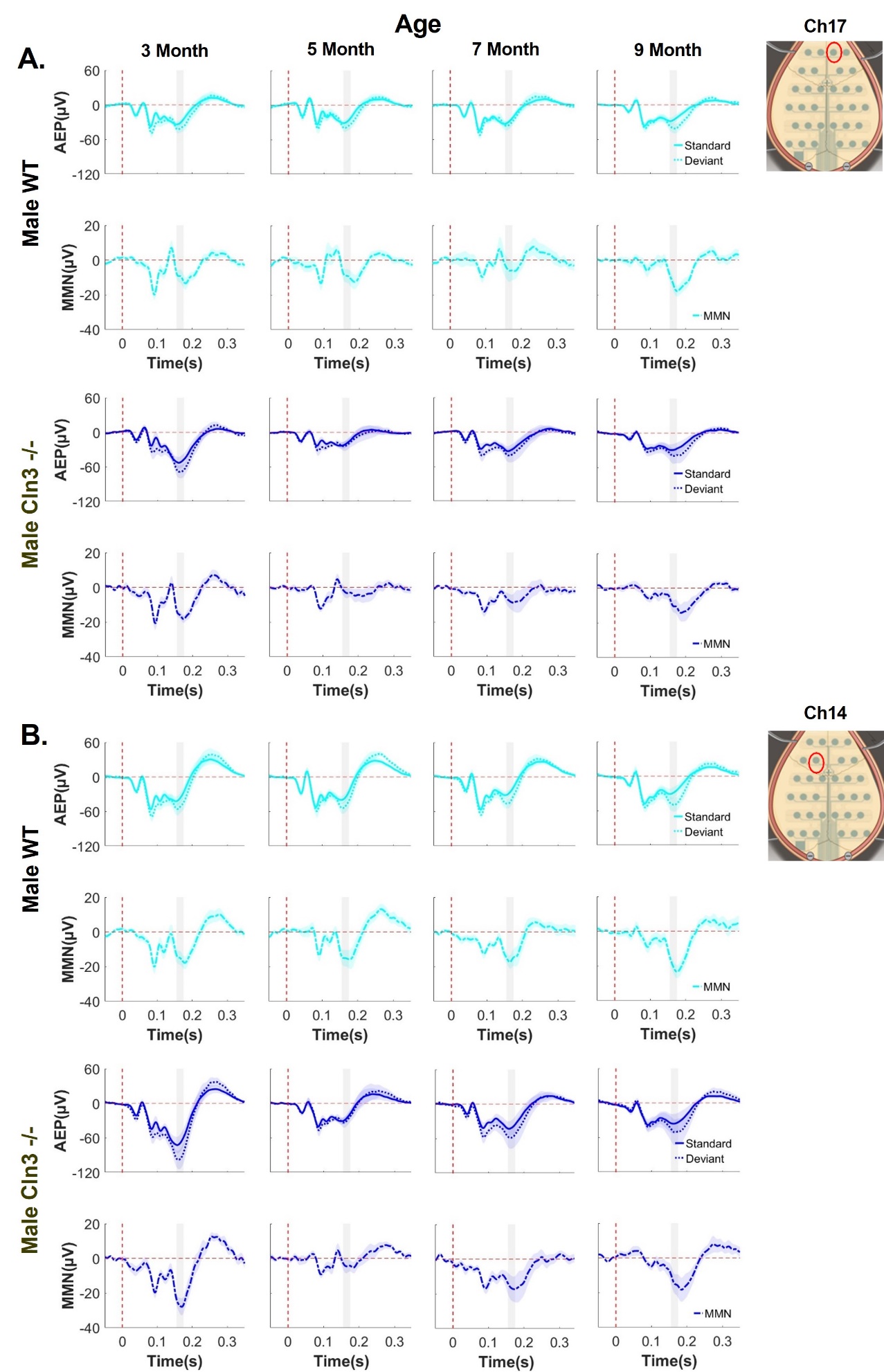


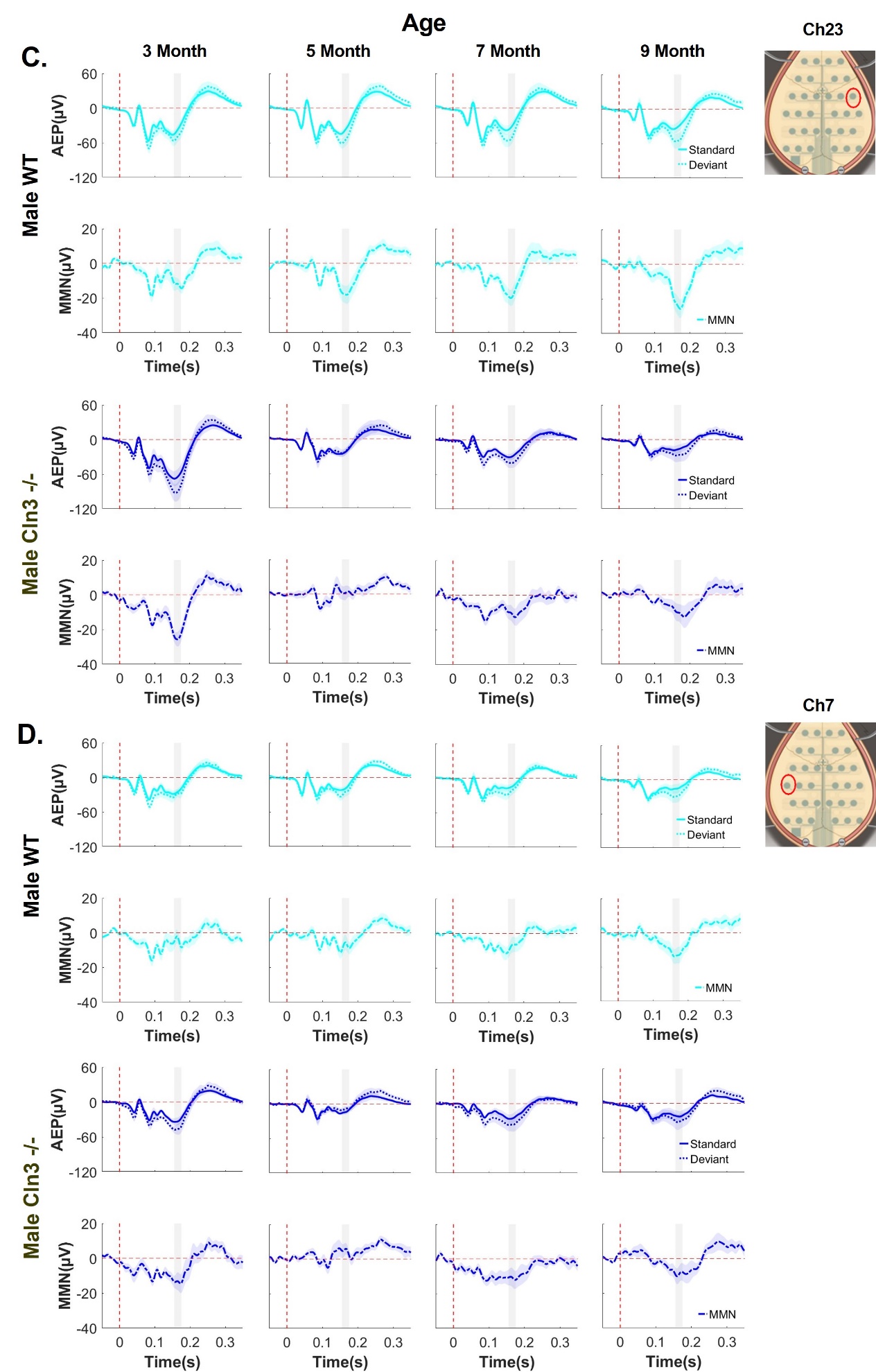


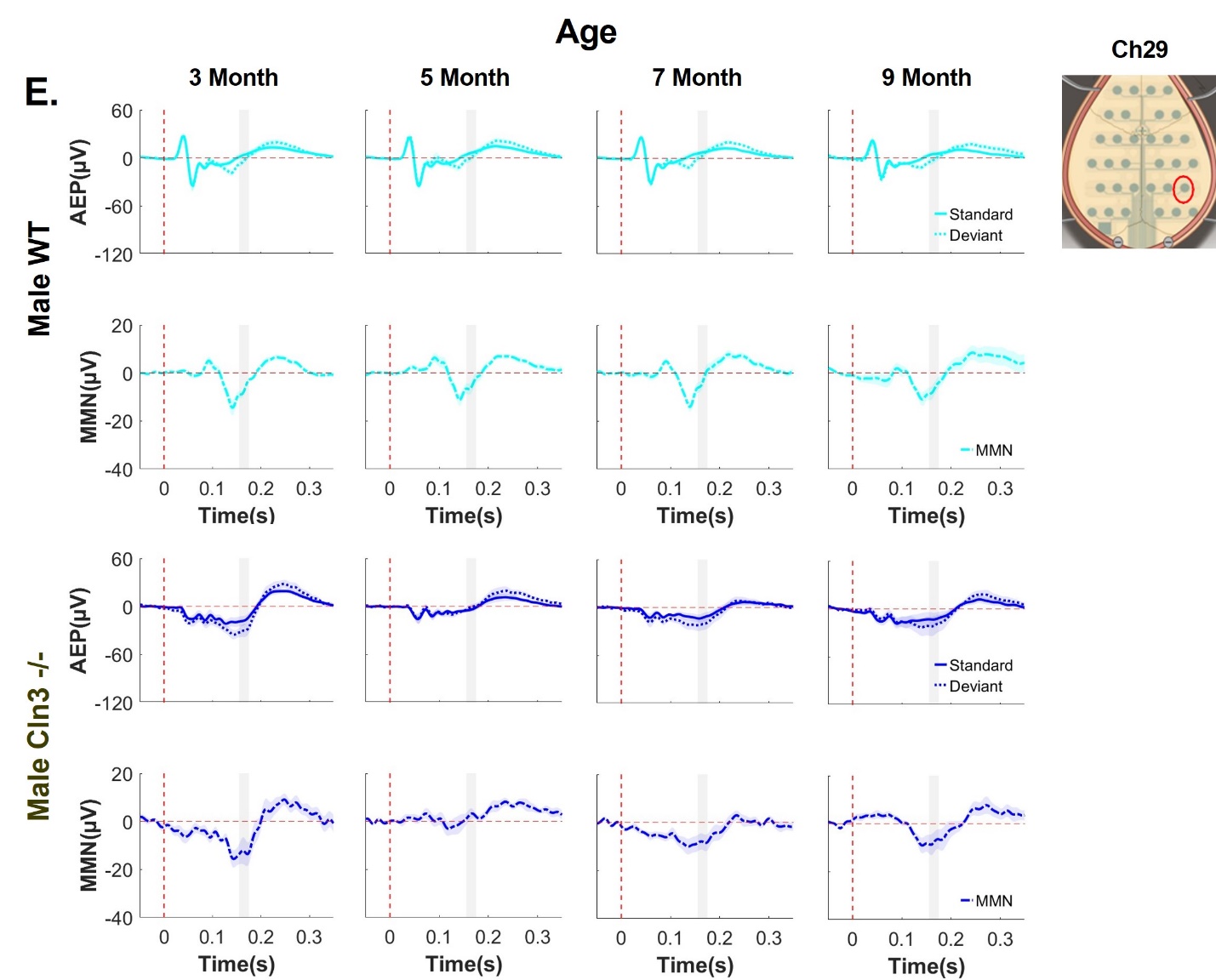


**Supplementary Figure 2. Male *Cln3-/-* mice showed disease progression-specific deficit of auditory duration MMN across different electrodes. *A-E,*** Group-averaged AEP and auditory duration MMN waveforms for male WT and *Cln3-/-* mice across different electrodes. ***A,*** electrode 17 (most anterior). ***B,*** electrode 14 (anterior). ***C,*** electrode 23 (central). ***D,*** electrode 7 (central). ***E,*** electrode 29 (posterior). Vertical dash red line indicates onset of stimuli and horizontal dash red line indicates 0. The gray rectangle indicates the MMN time window (160-180ms after stimulus onset). Standard AEP in solid line, deviant AEP in dotted line, and MMN in solid- dotted line. Male WT mice (n = 11 for 3-, 7- and 9-month, n = 14 for 5 month) in light blue, male *Cln3-/-* mice (n = 8 for 3-, 7- and 9-month, n = 11 for 5 month) in dark blue.


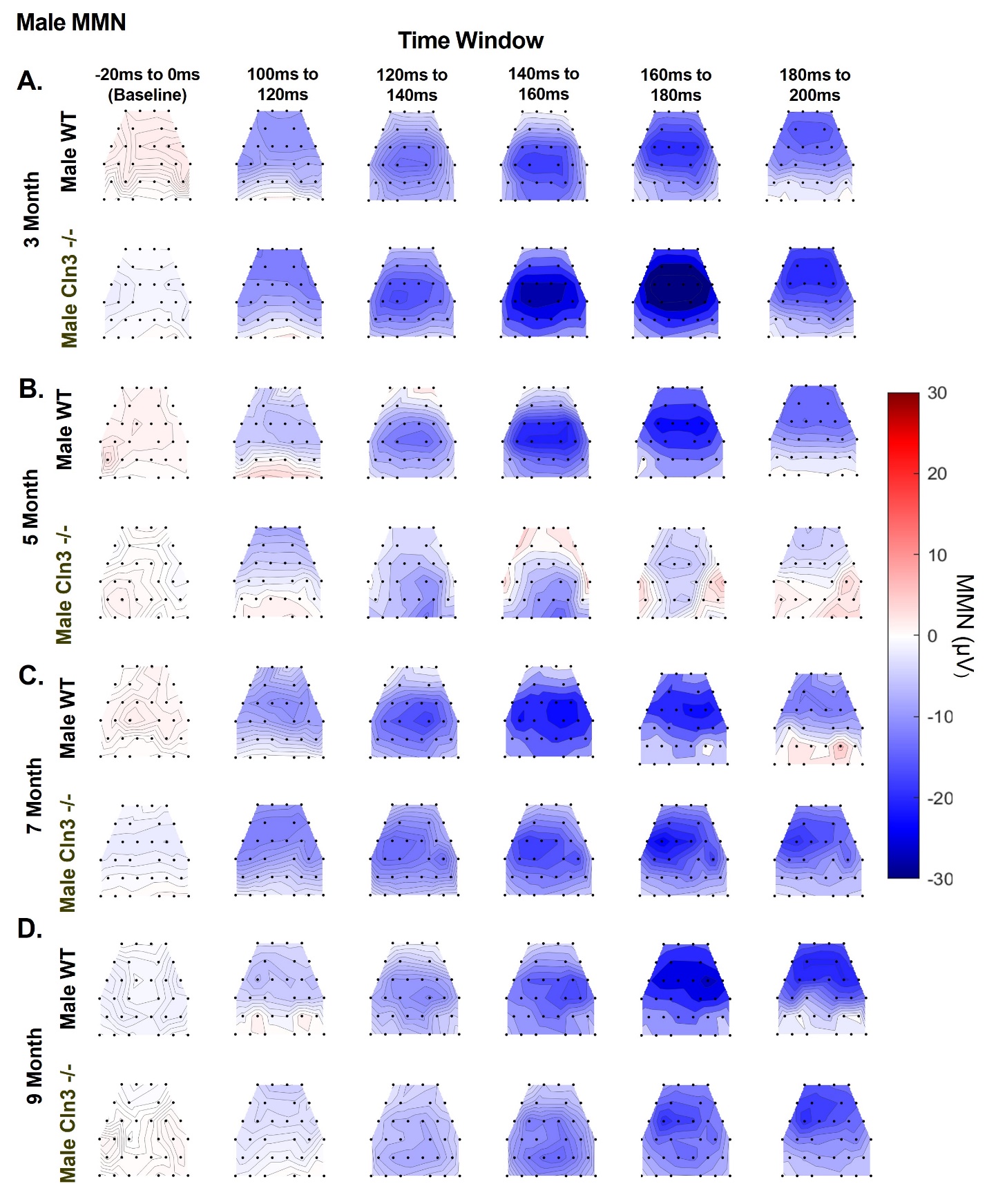


**Supplementary Figure 3. Male *Cln3-/-* mice showed late-age compensation of early-age auditory duration MMN deficits in spatial distribution.** Averaged topographical maps of auditory duration MMN in a 20ms time window from male WT (n = 11 for 3, 7, and 9 months, n = 14 for 5 months) and male *Cln3-/-* mice (n = 8 for 3, 7 and 9 months, n = 11 for 5 months). ***A,*** MMN topographical maps for 3-month-old mice. ***B,*** MMN topographical maps for 5-month-old mice. ***C,*** MMN topographical maps for 7-month-old mice. ***D,*** MMN topographical maps for 9 months old mice. Male *Cln3-/-* mice showed initial greater response, then decline and later recovery of auditory duration MMN in spatial distribution.


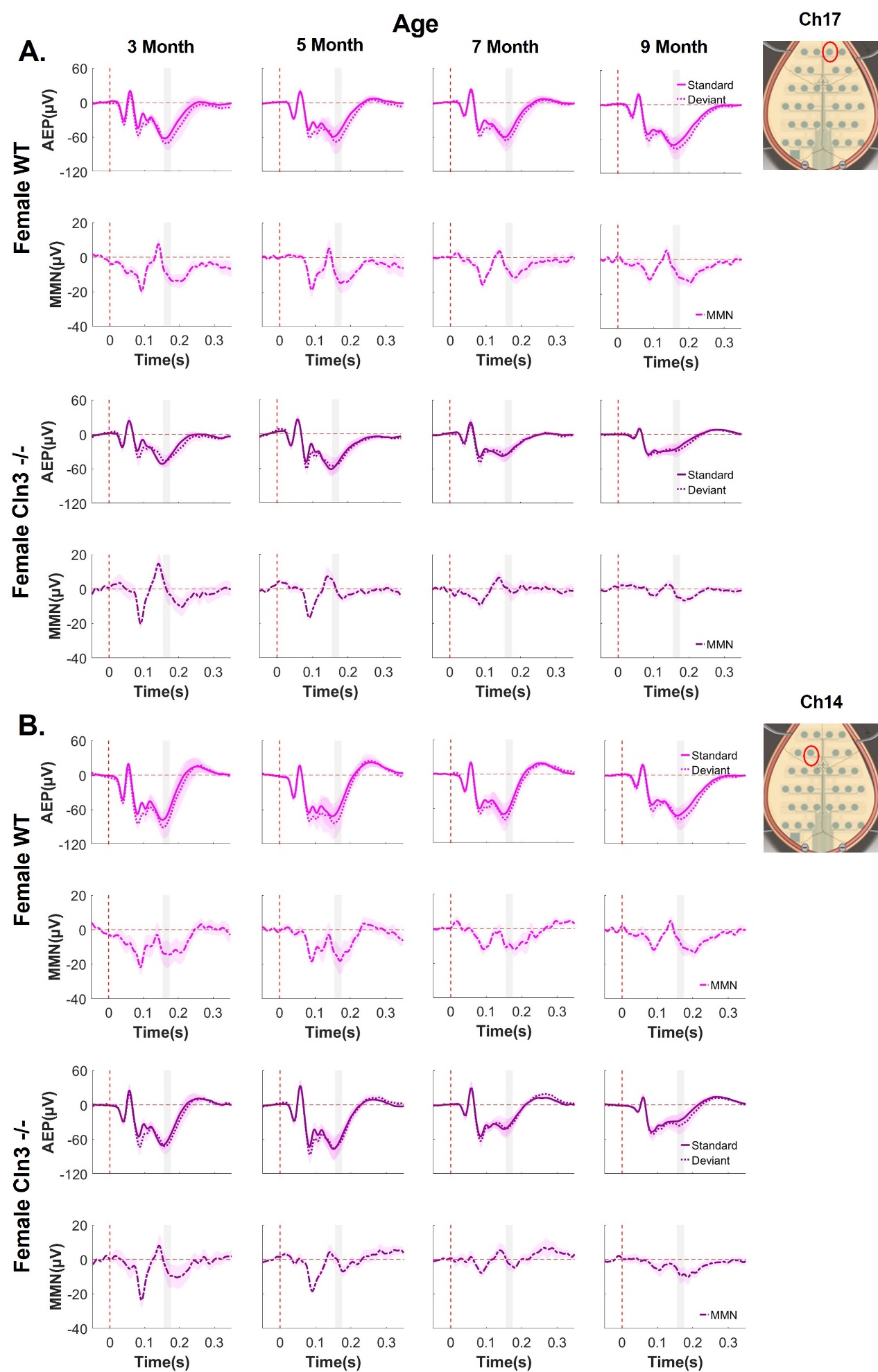


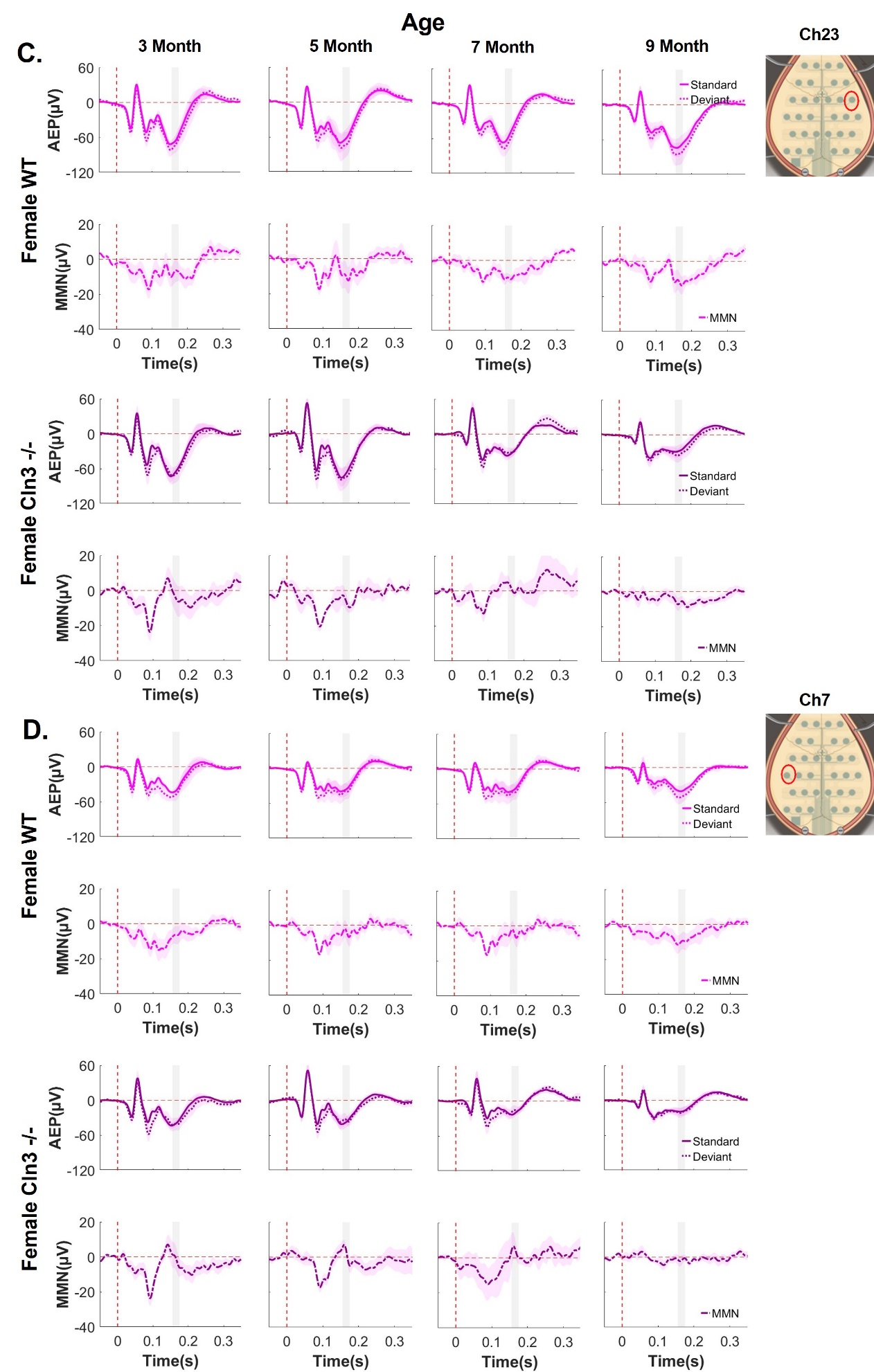


**
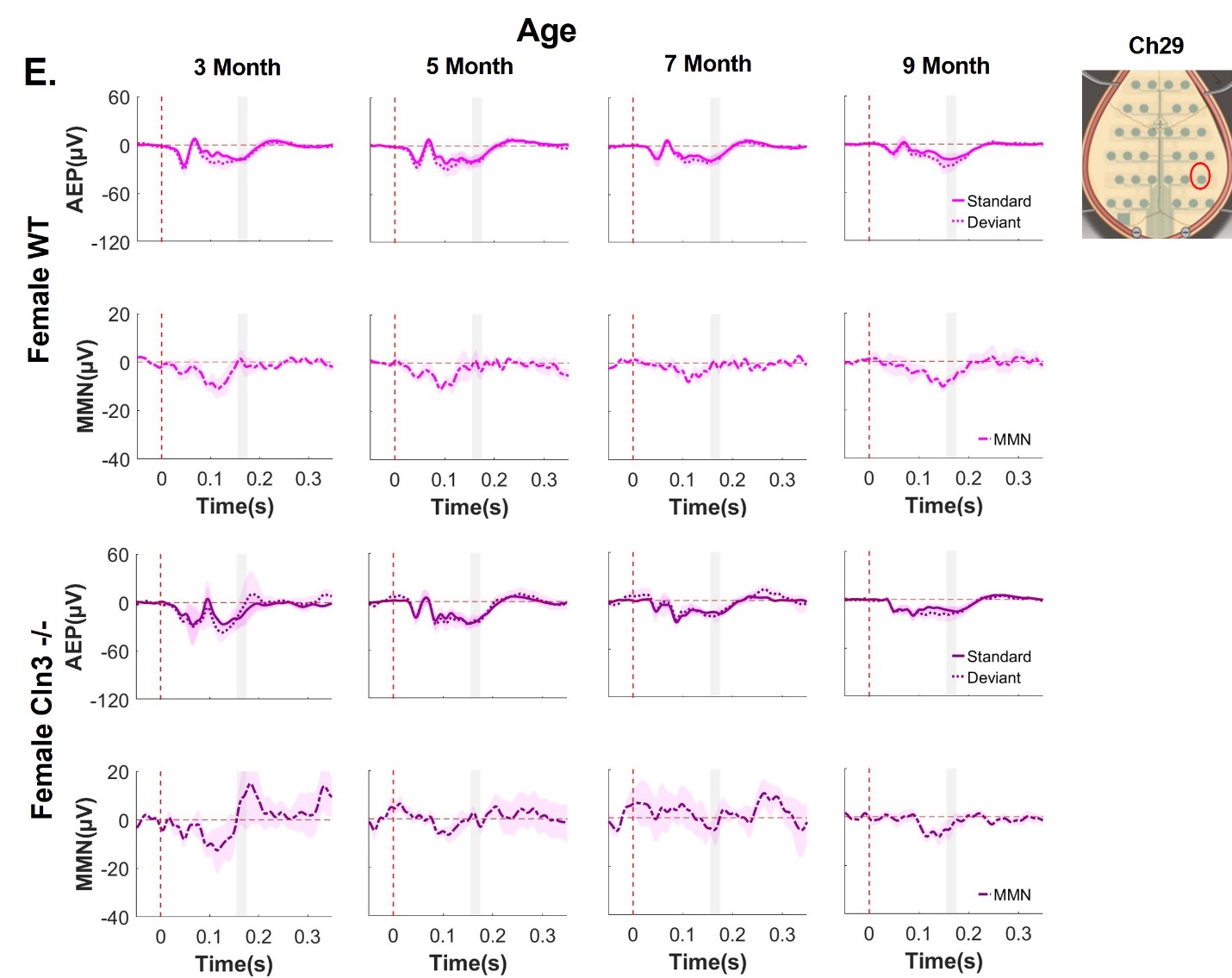
**

**Supplementary Figure 4. Female *Cln3-/-* mice showed a progressive decline in auditory duration MMN across different electrodes. *A-E,*** Group-averaged AEP and auditory duration MMN waveforms for female WT and *Cln3-/-* mice across different electrodes. ***A,*** electrode 17 (most anterior). ***B,*** electrode 14 (anterior). ***C,*** electrode 23 (central). ***D,*** electrode 7 (central). ***E,*** electrode 29 (posterior). The vertical dash red line indicates the onset of stimuli, and the horizontal dash red line indicates 0. The gray rectangle indicates the MMN time window (160-180ms after stimulus onset). Standard AEP in a solid line, deviant AEP in a dotted line, and MMN in solid- dotted line. Female WT mice (n = 6 for all ages) in light pink and female Cln3-/- mice (n = 7 for all ages) in dark pink.

**
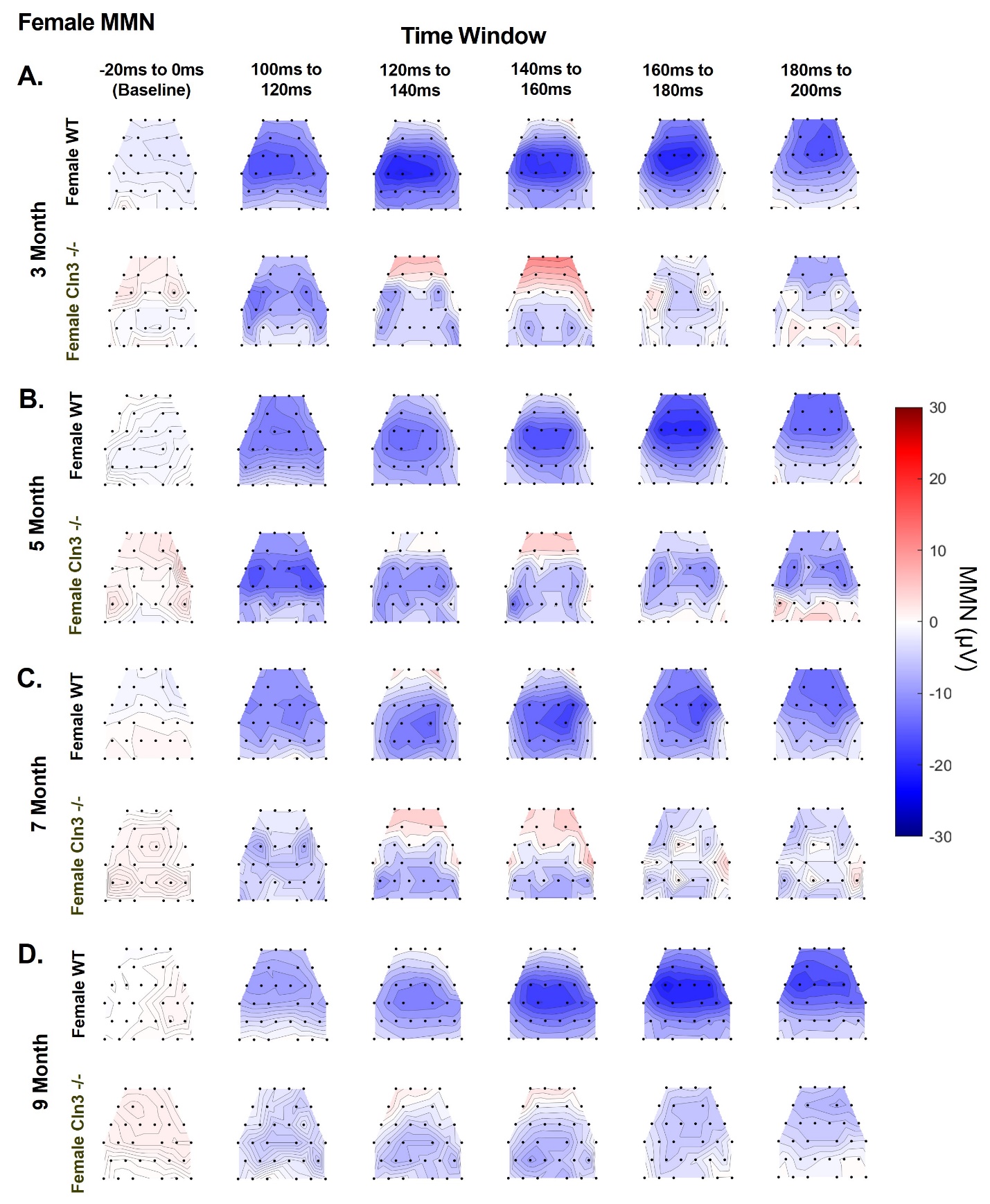
**

**Supplementary Figure 5. Female *Cln3-/-* mice showed consistent auditory duration MMN in spatial distribution.** Averaged topographical maps of auditory duration MMN in a 20ms time window from female WT (n = 6 for all ages) and female *Cln3-/-* mice (n = 7 for all ages). ***A,*** MMN topographical maps for 3-month-old mice. ***B,*** MMN topographical maps for 5-month-old mice. ***C,*** MMN topographical maps for 7-month-old mice. ***D,*** MMN topographical maps for 9-month-old mice. Female *Cln3-/-* mice showed consistent auditory duration MMN in spatial distribution.

**
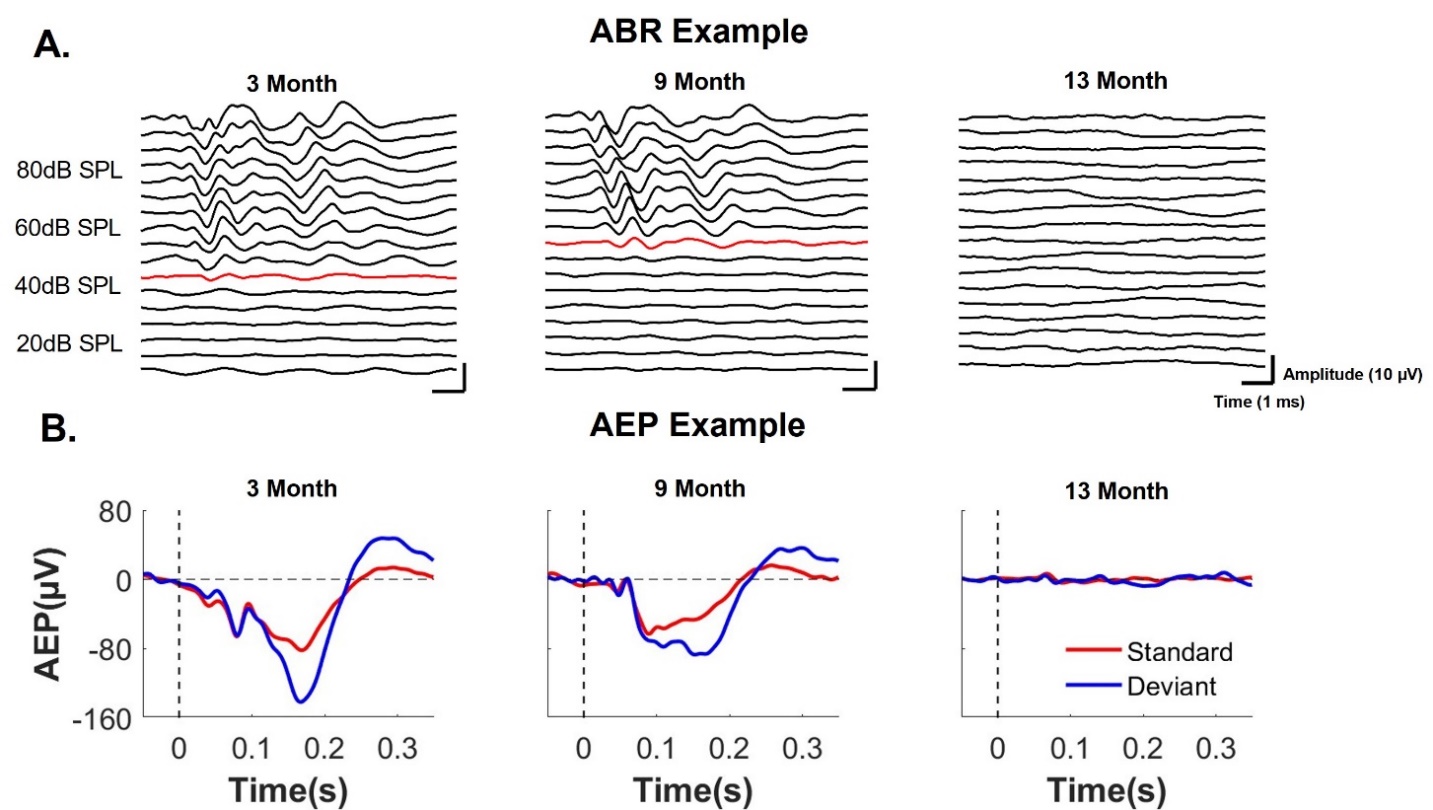
**

**Supplementary Figure 6. Animals with complete hearing loss had no identifiable ABR and AEP waveforms. *A,*** ABR waveforms from one 3-month-old, one 9-month-old, and one 13-month-old animal. There was no identifiable ABR waveform from the 13-month-old animal, suggesting a hearing threshold above 90dB SPL. ***B,*** group-averaged AEP waveforms for one 3-month-old, one 9-month-old, and one 13-month-old animal at Ch21. The vertical dash red line indicates the onset of stimuli, and the horizontal dash red line indicates 0. Standard AEP is in red and deviant AEP is in blue. Animals at younger ages showed clear AEPs with robust auditory duration MMN. There were no identifiable AEP waveforms for the 13-month-old animal. Complete hearing loss leads to the diminishment of ABRs and AEPs.


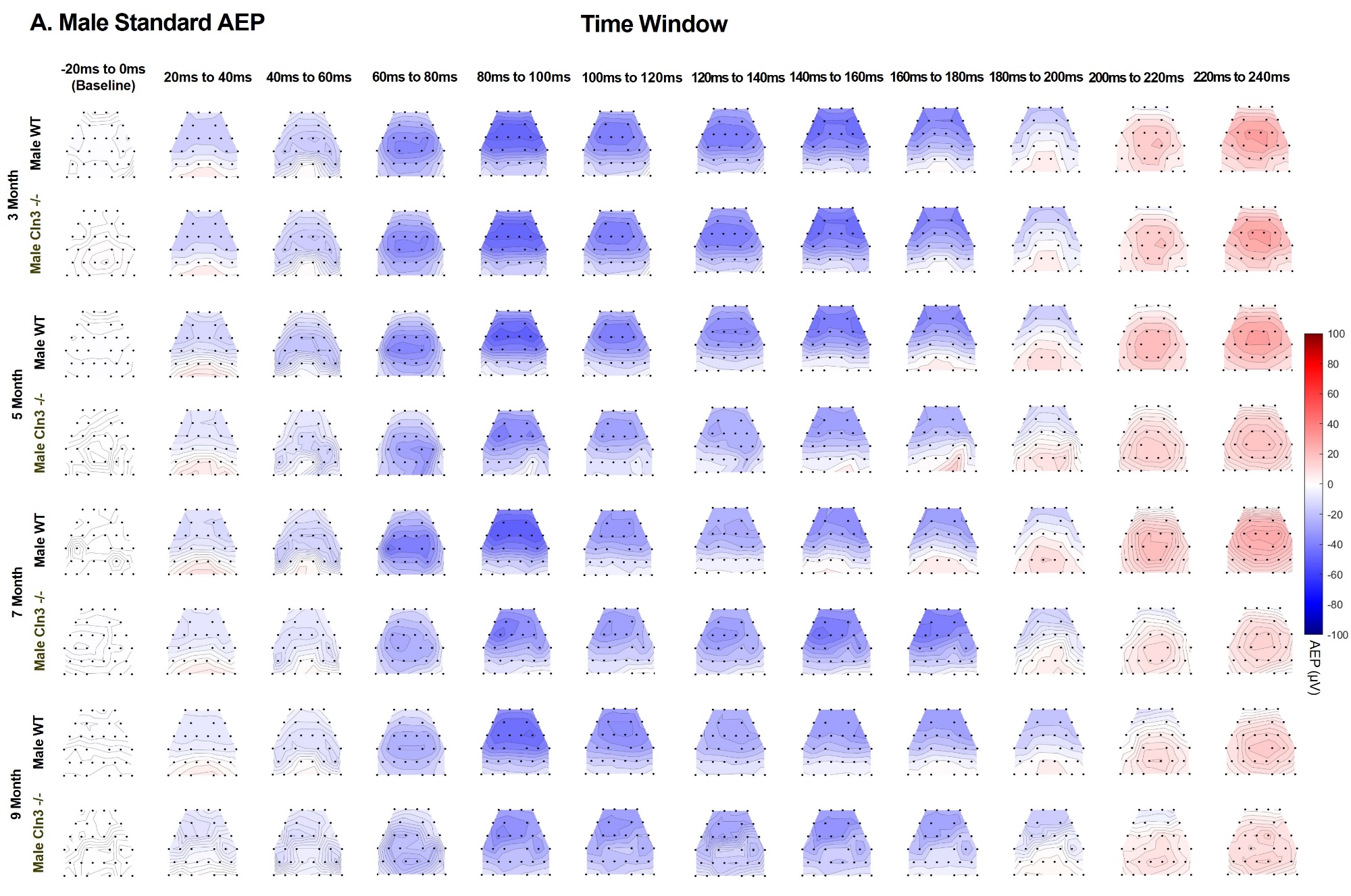


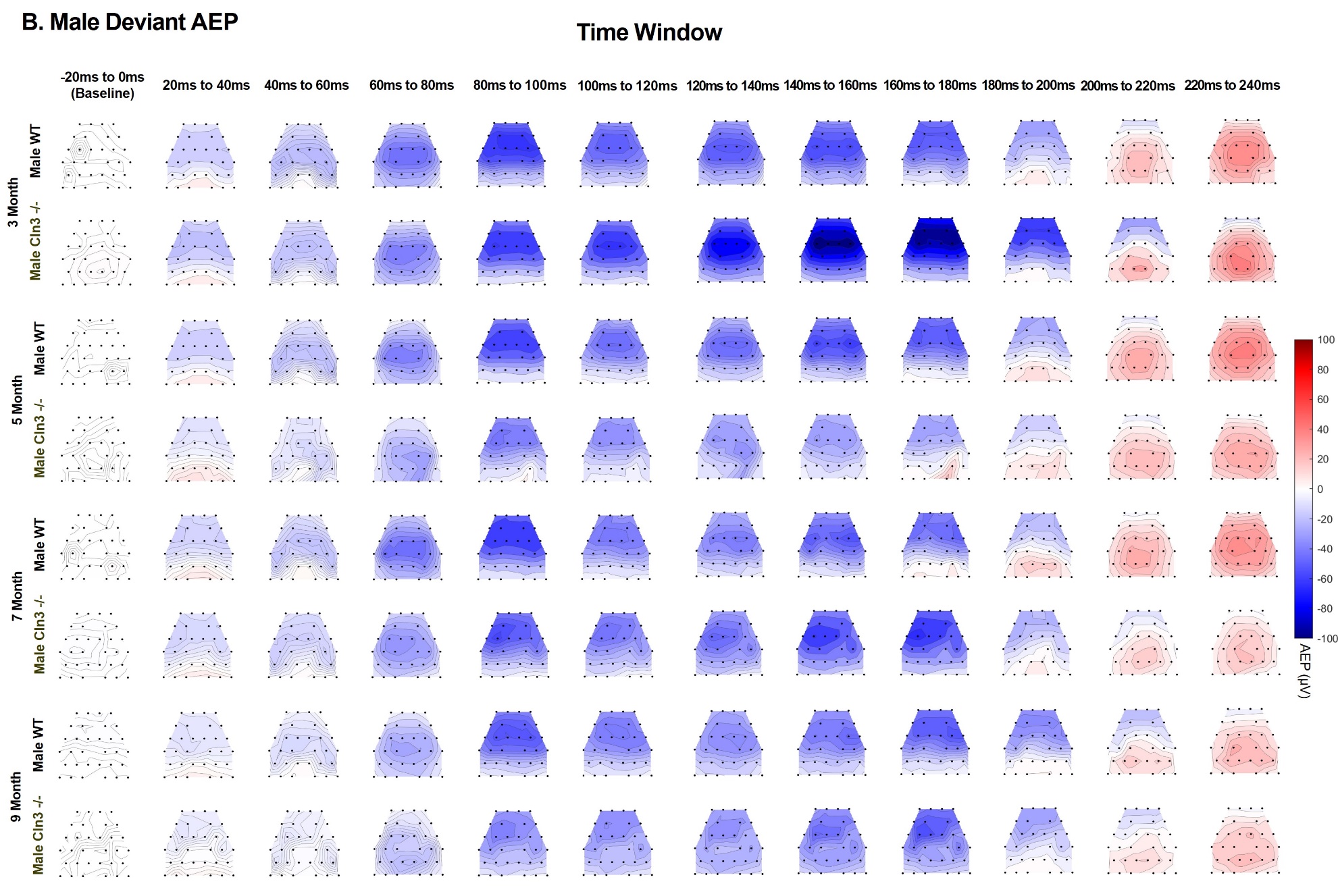


**Supplementary Figure 7. Male *Cln3-/-* mice showed late-age compensation of early-age AEP deficits in spatial distribution.** Averaged topographical maps of AEPs in a 20ms time window from male WT (n = 11 for 3, 7, and 9 months, n = 14 for 5 months) and male *Cln3-/-* mice (n = 8 for 3, 7, and 9 months, n = 11 for 5 months). ***A,*** topographical maps for male standard AEPs. ***B,*** topographical maps for male deviant AEPs. Male *Cln3-/-* mice showed initial greater response, then decline and later recovery of deviant AEPs in spatial distribution. Blue shows negativity and red shows positivity for AEP.


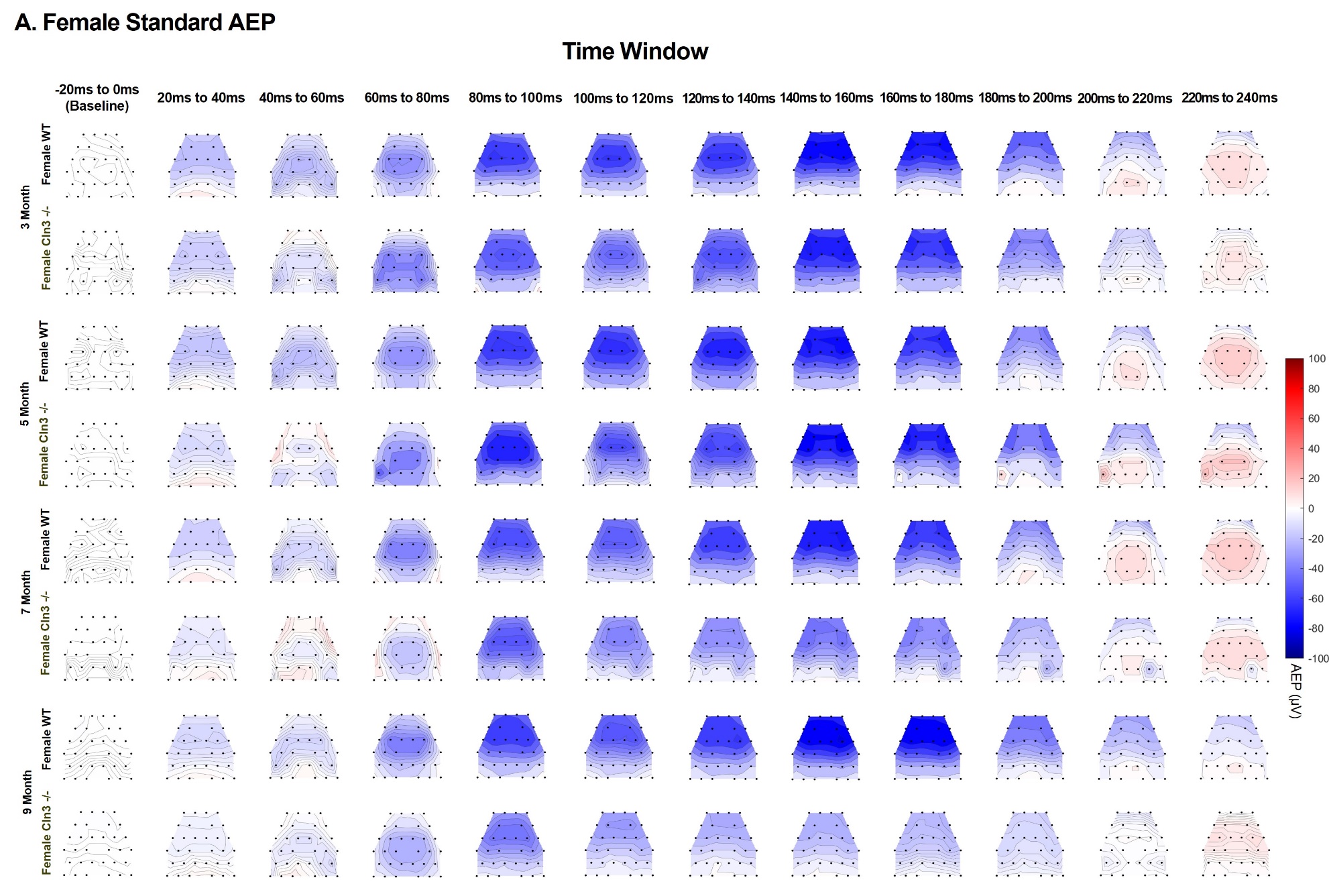


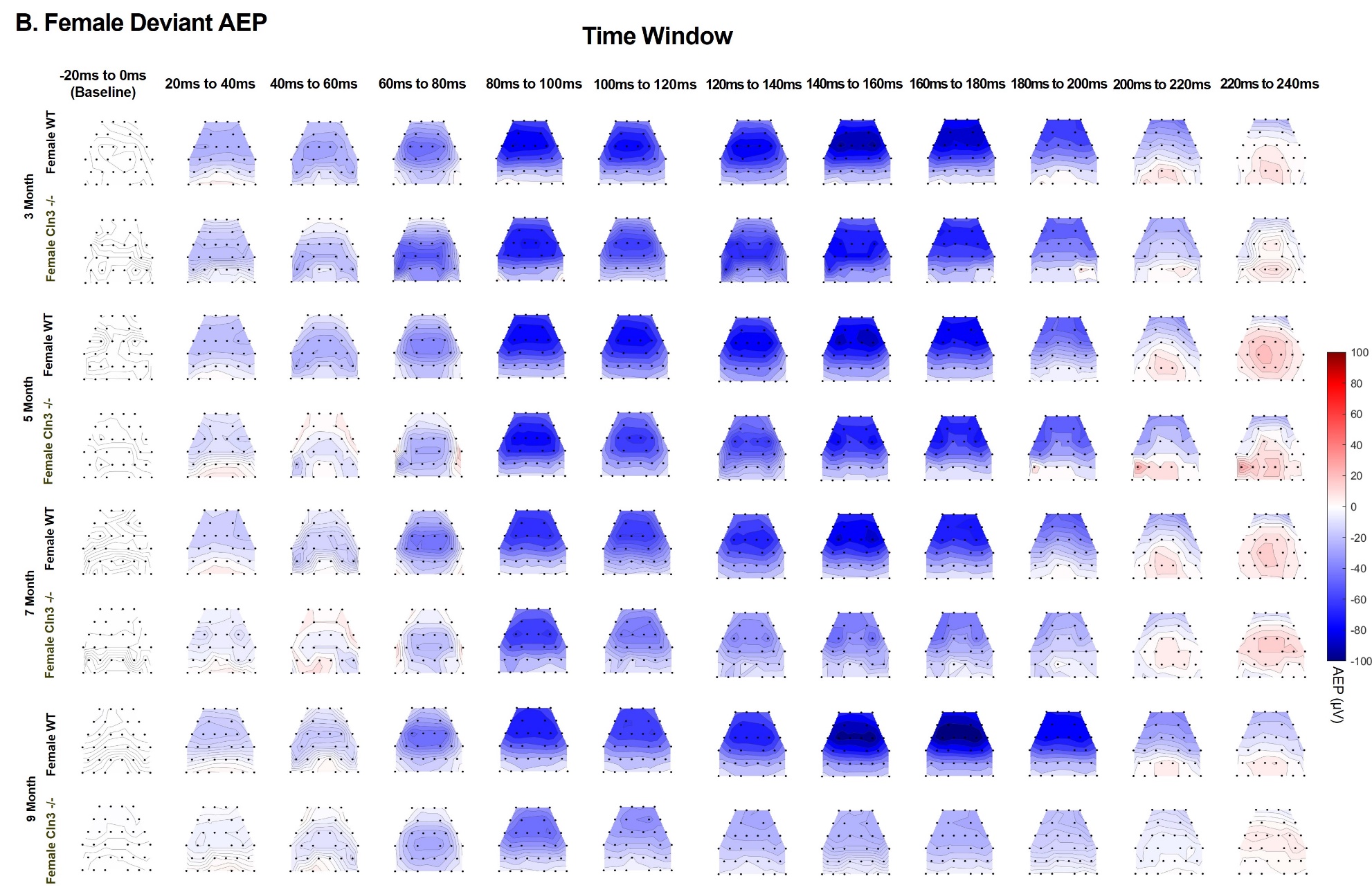


# **Supplementary Figure 8. Female Cln3-/- mice showed a progressive decline of AEPs in spatial distribution.** Averaged topographical maps of AEPs in a 20ms time window from female WT (n = 6 for all ages) and female Cln3-/- mice (n = 7 for all ages). ***A,*** topographical maps for female standard AEPs. ***B,*** topographical maps for female deviant AEPs. Female Cln3-/- mice showed a progressive decline of AEPs in spatial distribution. Blue shows negativity and red shows positivity for AEPs.
